# Immune-Related Adverse Events Associated With Immune Checkpoint Inhibitors for Advanced Non-small Cell Lung Cancer: A Network Meta-Analysis of Randomized Clinical Trials

**DOI:** 10.3389/fphar.2021.686876

**Published:** 2021-10-25

**Authors:** Weidong Zhang, Jingjing Gu, Chunming Bian, Guanhong Huang

**Affiliations:** ^1^ Center of General Surgery, The Xixi Hospital of Hangzhou, Zhejiang, China; ^2^ The Second People’s Hospital of Lianyungang, The Affiliated Hospital of Bengbu Medical College, Lianyungang, China

**Keywords:** immune-related adverse events, non-small cell lung cancer, network meta-analysis, immune checkpoint inhibitor, randomized clinical trial

## Abstract

**Objective:** This network meta-analysis will provide a complete toxicity profile, toxicity profile, and safety ranking of immune checkpoint inhibitors (ICIs) for treatment of advanced non-small cell lung cancer (NSCLC).

**Methods:** We found 12 phase II or III randomized clinical trials (RCTs) including 8,453 patients with NSCLC by searching Pubmed, Embase, and ClinicalTrials.gov. Risk ratios (RRs) and 95% confidence interval (CI) were used to compare the rate of immune-related adverse events (irAEs) for different ICIs-based treatments using pairwise and network meta-analysis with random effects.

**Results:** For dermatologic irAEs, the corresponding ranking of incidences of the seven groups from high to low was: nivolumab + ipilimumab (97.4%), pembrolizumab (80.1%), nivolumab (67.1%), pembrolizumab + platinum (43.3%), atezolizumab + platinum (39.9%), durvalumab (17.5%), platinum-based chemotherapy (4.7%). For colitis, the corresponding ranking of incidences of the six groups from high to low was: atezolizumab + platinum (77.1%), nivolumab (67.3%), pembrolizumab (60.5%), durvalumab (45.2%), pembrolizumab + platinum (41.4%), platinum-based chemotherapy (8.5%). For endocrine irAEs, the corresponding ranking of incidences of the seven groups from high to low was: nivolumab + ipilimumab (79.1%), durvalumab (69.1%), pembrolizumab (61.9%), atezolizumab + platinum (60.4%),nivolumab (45.7%), pembrolizumab + platinum (33.5%), platinum-based chemotherapy (0.3%). For pneumonitis, the corresponding ranking of incidences of the seven groups from high to low was: pembrolizumab (99.3%), pembrolizumab + platinum (65.1%), durvalumab (62.2%), atezolizumab + platinum (56%), nivolumab (35.9%), platinum-based chemotherapy (18.1%),atezolizumab (13.3%). For hepatitis, the corresponding ranking of incidences of the six groups from high to low was: pembrolizumab (71.2%), pembrolizumab + platinum (64.3%), durvalumab (56.4%), atezolizumab + platinum (53.8%), nivolumab (44.5%), platinum-based chemotherapy (9.8%).

**Conlusion:** In addition to platinum-based chemotherapy, durvalumab for dermatologic and liver irAEs, pembrolizumab for gastrointestinal irAEs, pembrolizumab + platinum for endocrine irAEs, and atezolizumab for pneumonitis may be associated with lower rates of irAEs than other immune-based regimens. Nivolumab + ipilimumab for dermatologic and endocrine irAEs, atezolizumab + platinum for colitis, and pembrolizumab for pneumonitis and hepatitis may be associated with higher rates of irAEs.

## Introduction

According to the latest statistics, lung cancer is still the leading cause of cancer deaths, although the incidence of cancer has declined in recent years (Goldstraw et al., 2016; Siegel et al., 2020). Nearly 70% of patients with lung cancer are diagnosed with locally advanced or metastatic disease (Torre et al., 2016), and the 5-years survival rate is only 5% (Molina et al., 2008; Bironzo and Di Maio, 2018). However around 80–85% of lung cancer cases are classified as NSCLC (Torre et al., 2015). Platinum-based chemotherapy remains the standard first-line treatment for NSCLC without target gene mutations, with a response rate of only 15% (Sandler et al., 2006; Scagliotti et al., 2008).

In recent years ICIs, which constitute an inhibitory pathway detected in a variety of malignant tumors, have opened a new era of cancer treatment (Ettinger et al., 2017; Hanna et al., 2017). Tumor cells form immune escape through immune checkpoints, however ICIs are to prevent the immune escape caused by the tumor cells by combining with the immune checkpoint, thus enabling the immune cells to resume their killing effect on the tumor cells. Currently, programmed cell death receptor 1(PD-1) and cytotoxic T lymphocyte-associated antigen-4(CTLA4) are widely studied immune checkpoints. PD-1 is a type I transmembrane glycoprotein (He et al., 2015), which is an inhibitory receptor of T cells. It can bind and interact with the specific ligands programmed cell death ligand-1(PD-L1) and programmed cell death ligand-2 (PD-L2). Tumor cells can activate PD-1, thereby promoting it to bind to PD-L1 and PD-L2 on the surface of antigen presenting cells, inhibiting the proliferation of effector T cells and preventing T cells from recognizing dangerous molecules in a timely and effective manner, thus enabling tumor cells to evade the pursuit of immune cells. PD-1/PD-L1 inhibitors promote the activation and proliferation of T cells by inhibiting the expression of PD-1/PD-L1, thus stimulating the killing of tumor cells (Pedoeem et al., 2014). CTLA-4 is a leukocyte differentiation antigen and a transmembrane receptor on T cells. It shares the B7 molecular ligand with CD28, while CTLA-4 induces T cells to be inreactive after binding to B7 molecule, and participates in the negative regulation of immune response. However CTLA-4 inhibitors can inhibit the molecule CTLA-4, allowing T cells to proliferate and attack tumor cells. Data from a series of randomized clinical trials suggest that ICIs alone or in combination as first-line treatment for advanced NSCLC patients provides better clinical benefits and fewer side effects than conventional platinum-based chemotherapy (Corey et al., 2016; Reck et al., 2016; Carbone et al., 2017; Barlesi et al., 2018; Gandhi et al., 2018; Paz-Ares et al., 2018; Hellmann et al., 2019; Tony et al., 2019; West et al., 2019; Jotte et al., 2020; Naiyer et al., 2020; Roy et al., 2020). However, ICIs enhance self immune functions against cancer cells through a unique mechanism that blocks negative regulators expressed on immune or tumor cells, while yielding higher rates of irAEs than platinum-based chemotherapy (Remon et al., 2020). According to statistics, the occurrence of grade 3 or higher irAEs varied from 8 to 10% among patients with advanced NSCLC receiving ICIs (Remon et al., 2020). Common target organs of irAEs included dermatologic irAEs (pruritus and rash), endocrine irAEs (hypothyroidism and hyperthyroidism), colitis, pneumonitis and hepatitis (Wang et al., 2018). Any of these irAEs, if not properly treated and managed in clinical practice, irAEs will lead to treatment termination, failure, and may even be life-threatening. Therefore, it was very necessary for clinicians to better grasp the irAEs most likely to be caused by each treatment regime, and to prevent, detect and treat them as early as possible. However, which treatment regime was more likely to induce irAEs was controversial.

Here, to help clinicians improve early prediction, early detection and early treatment of irAEs, a network meta-analysis was conducted to compare the incidences of irAEs and rank the safety of ICI + chemotherapy, ICI alone, and dual ICIs combination.

## Methods

### Data Sources and Searches

This network meta-analysis (NMA) was based entirely on the preferred reporting items for systematic reviews and meta-analysis (PRISMA) (Liberati et al., 2009; Hutton et al., 2015) and PRISMA extended guidelines for an NMA. RCTs on ICIs versus platinum-based chemotherapy as the first-line treatment of advanced NSCLC from 2015 to 2020 were searched in PubMed, Embase. To search for data not explicitly given in the RCTs, the National Institutes of Health ongoing Trial Registry (Clinicaltrials.gov) was also searched. A combination of MeSH and free-text words was searched according to the PICOS principle.Search terms and their combinations used in the search strategy included (PD-1 OR PD-L1 OR CTLA-4 inhibitor OR nivolumab OR pembrolizumab OR durvalumab OR atezolizumab OR ipilimumab) AND (Non-Small-Cell Lung Carcinoma OR Non-Small Cell Lung Cancer) AND chemotherapy AND (randomized controlled trial). Two independent reviewers (Jingjing Gu and Weidong Zhang) conducted a preliminary screening of the searched topics and abstracts, and if they did not meet the criteria, further read the full text, and all references were evaluated as potentially relevant articles.

### Inclusion and Exclusion Criteria

Inclusion criteria for RCTs of this NMA were as follows: 1) study type: only Phase II or III double blind RCTs for advanced NSCLC; 2) participants: included patients that were pathologically diagnosed as advanced NSCLC; 3) experimental group: patients were treated with immunotherapy alone or an immune-based combination as first-line treatment, control group: patients were treated with only platinum-based chemotherapy as first-line treatment; 4) outcome indicators: there was at least one irAE in the RCTs or searched on Clinicaltrials.gov.

Exclusion criteria: non-English articles, studies without valid data, reviews, meta-analyses, editorials, commentary letters, repeat studies.

### Data Extraction

By reading the titles, abstracts and full texts, the two authors screened out the articles that met the predetermined inclusion criteria. 1) Trial information, including first author, study year, trial id; 2) Stage information, study endpoint, and sample size of treatment; 3) Patient characteristics at baseline included median age, sex, and the numbers of patients with dermatologic irAEs, colitis, endocrine irAEs, pneumonitis and hepatitis (grade 1–5). And the two authors checked the extracted data before data analysis.

### Risk-Of-Bias Assessment

**FIGURE 1:**
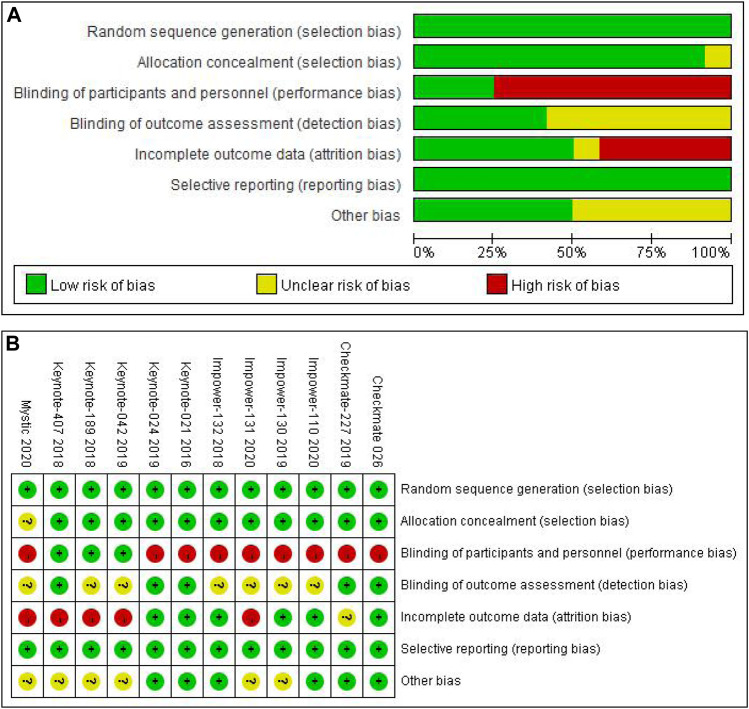
The Cochrane risk bias assessment tool was used to evaluate bias from seven key sources: 1. Random sequence generation; 2. Allocation concealment; 3. Blindness of subjects and researchers; 4. Blindness of outcome evaluation; 5. Incomplete data; 6. Selective reporting of results; 7. Other biases. Green represents low risk, yellow represents unclear risk, and red represents high risk. **(A)** Risk of bias graph; **(B)** Risk of bias summary.

The Cochrane risk bias assessment tool (Higgins and Green, 2011) (Figure1)were used to evaluate the quality of the literature by two reviewers (Jingjing Gu and Weidong Zhang). The seven major sources of biases (random sequence generation, allocation concealment, blinding of participants and personnel, blinding of outcome assessment, incomplete outcome data, selective reporting and other bias) were classified into three grades including “yellow represents unclear risk”, “green represents low risk” and “red represents high risk” which were assessed using the Cochrane risk bias assessment tool.

### Outcome Measures

The study endpoints were any grade of dermatologic irAEs including pruritus and rash, colitis, endocrine irAEs including hypothyroidism and hyperthyroidism, pneumonitis and hepatitis. Review criteria were based on the National Cancer Institute Common Terminology Criteria for Adverse Events (Basch et al., 2016). The probability of associated adverse events for each treatment regimen was assessed by the combined RRs and 95% CI.

### Data Synthesis and Statistical Analysis

The loops to illustrate the network geometry were generated using Stata13.0. In this NMA, summary RRs and 95% confidence intervals were used as the results of the effect size of ICI-based drugs on the risk of irAEs in NSCLC patients. RR greater than 1 represented a low probability of irAEs in the control group. Markov chain Monte Carlo (MCMC) methods were used to establish random-effects and consistency models calculating the RR and 95% confidence intervals within the Bayesian framework by using R (version 4.0.1) (CoreTeam 2019, Vienna, Austria) and JAGS (version 4.3.0) with the package “getmtc” (version 0.8.2) (Salanti et al., 2008; Sutton et al., 2008). Heterogeneity of the included studies was assessed by I^2^ statistics. I^2^ values below 25%, between 25 and 50%, and above 50% represented low, medium, and high heterogeneity, respectively (Mills et al., 2013). If the heterogeneity is low, the fixed effects model would be selected, otherwise the random effects model would be selected. In order to explore the inter-study heterogeneity of each outcome comparison, the values of different parameters of a log-normal distribution were fitted as a prior distribution (Higgins et al., 2003). To obtain the posterior distribution, 10,000 burn-ins and 50,000 iterations of 4 each chain and a thinning interval of 10 were generated for each outcome by using MCMC methods. We judged whether each MCMC chain reached a stable and good iteration during the calculation process through the Brookse-Gelmane-Rubin diagnostic plot with a cut-off value of 1, so as to determine whether the degree of convergence of the model was satisfactory. The surface under the cumulative ranking curve (SUCRA) metric was a tool to rank the probabilities of irAEs of each treatment and identify the best treatment. The SUCRA value was ranged from 0 to 1, the closer to 1 this value approached, the higher its incidence of an immune-related adverse event was in this network meta-analysis (Salanti et al., 2011).A funnel plot was used to assess the publication bias and symmetrical distribution in funnel plot suggests no publication bias (Higgins et al., 2012). Sensitivity analysis was performed to assess the stability of the results which were considered stable if there was significant consistency between direct and indirect results (Dias et al., 2010). We conducted a inconsistency test (that is, the comparison of the differences between direct and indirect comparisons) using the nodal analysis and *p* > 0.05 indicated that there is no inconsistency (Sterne and Egger, 2001; Furukawa et al., 2016).

## Results

### Literature Search Results and Study Characteristics

**FIGURE 2:**
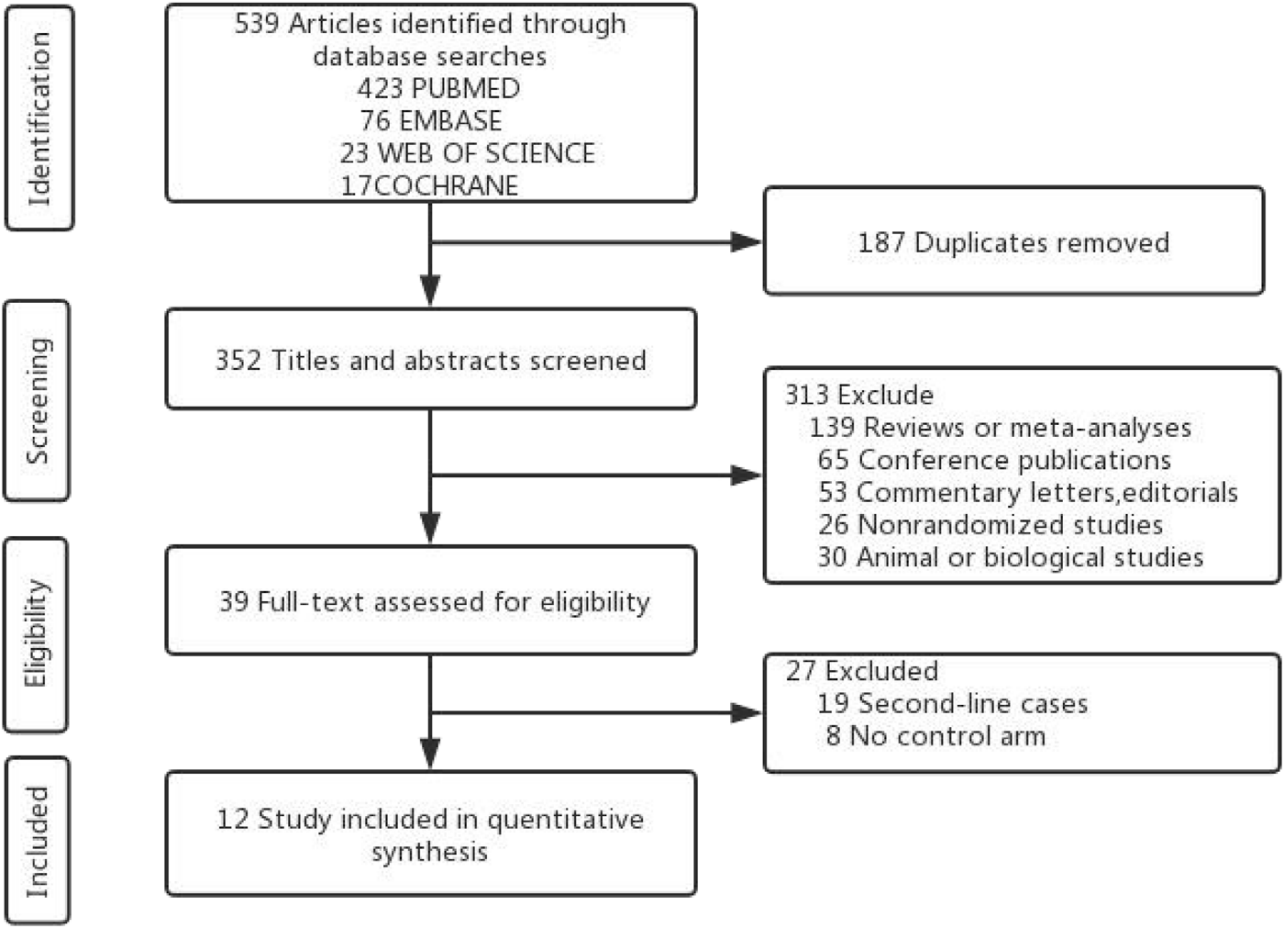
Flowchart of the systematic search PRISMA flow diagram.

**FIGURE 3:**
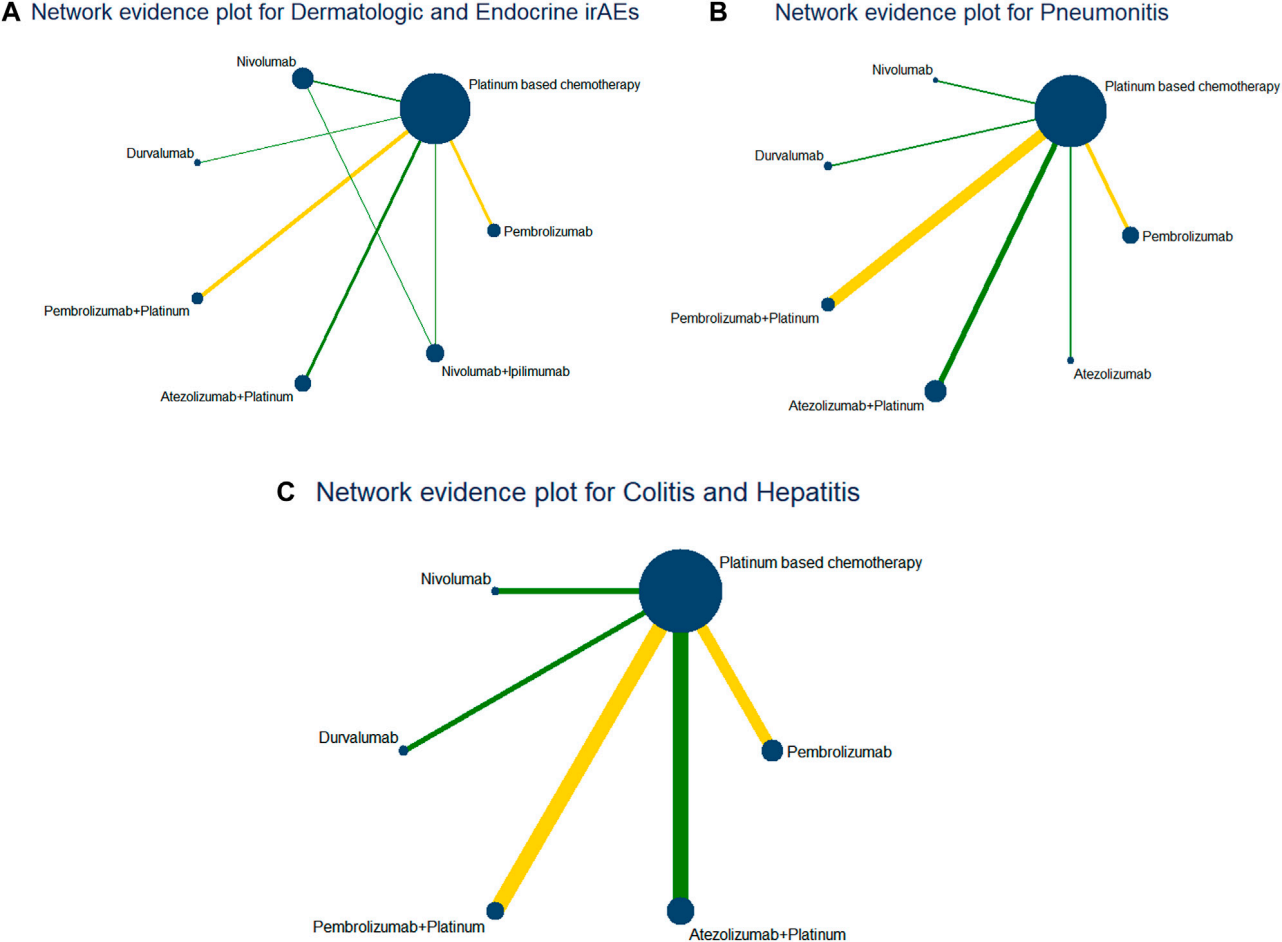
Network diagrams of comparisons on all outcomes in this network meta-analysis. **(A)** Comparisons on dermatologic and endocrine irAEs in patients with advanced NSCLC. **(B)** Comparisons on pneumonitis in patients with advanced NSCLC. **(C)** Comparisons on colitis and hepatitis in patients with advanced NSCLC.(The circles represent treatment regimens and the size of each circle represents the number of participants, while the yellow line represents double-blind RCTs and the green is not blind.)

Through the literature search, 539 studies were initially retrieved. After removing duplicates, 352 studies were used to filter through screening titles and abstracts, then 39 studies were assessed by screening full text. Finally, 12 RCTs (Reck et al., 2016; Corey et al., 2016; Carbone et al., 2017; Barlesi et al., 2018; Paz-Ares et al., 2018; Gandhi et al., 2018; Hellmann et al., 2019; Tony et al., 2019; West et al., 2019; Roy et al., 2020; Jotte et al., 2020; Naiyer et al., 2020) including 8,453 patients with advanced NSCLC were considered eligible for inclusion in this network meta-analysis. The literature retrieval strategy is shown in Figure 2. The included RCTs involved 1 Phase Ⅰ/Ⅱ trial and 11 Phase III trials and described eight treatment regimes (pembrolizumab, nivolumab, durvalumab, atezolizumab, pembrolizumab + platinum, atezolizumab + platinum, nivolumab + ipilimumab and platinum-based chemotherapy). The Network diagrams of comparisons on all outcomes in this network meta-analysis are presented in Figure 3. The dermatologic irAEs (pruritus and rash) and endocrine irAEs (hypothyroidism and hyperthyroidism) involved seven different treatment regimens (pembrolizumab, nivolumab, durvalumab, pembrolizumab + platinum, atezolizumab + platinum, nivolumab + ipilimumab and platinum-based chemotherapy) in 11 studies (Reck et al., 2016; Corey et al., 2016; Carbone et al., 2017; Paz-Ares et al., 2018; Gandhi et al., 2018; Barlesi et al., 2018; Tony et al., 2019; West et al., 2019; Hellmann et al., 2019; Naiyer et al., 2020; Jotte et al., 2020) (Figure 3A).The pneumonitis involved seven different treatment regimens (pembrolizumab, nivolumab, durvalumab, atezolizumab, pembrolizumab + platinum, atezolizumab + platinum, nivolumab + ipilimumab and platinum-based chemotherapy) in 11 studies (Reck et al., 2016; Corey et al., 2016; Carbone et al., 2017; Paz-Ares et al., 2018; Gandhi et al., 2018; Barlesi et al., 2018; West et al., 2019; Tony et al., 2019; Naiyer et al., 2020; Jotte et al., 2020; Roy et al., 2020) (Figure3B). Colitis and hepatitis involved six different treatment regimens (pembrolizumab, nivolumab, durvalumab, atezolizumab, pembrolizumab + platinum, atezolizumab + platinum and platinum-based chemotherapy) in 10 studies (Corey et al., 2016; Reck et al., 2016; Carbone et al., 2017; Barlesi et al., 2018; Gandhi et al., 2018; Paz-Ares et al., 2018; Tony et al., 2019; West et al., 2019; Jotte et al., 2020; Naiyer et al., 2020) (Figure3C). Key features of all the studies are shown in Table 1 and Table 2.

**TABLE 1:** General characteristics of the included randomised control trials for this network meta-analyses (Abbreviations: MN,multinational; NA, not applicable.)

First author, year	Study ID	Region	Trial phase	Trail number	Experimental group	Control group
Carbone 2017	Checkmate 026	MN	II	423	Nivolumab 3 mg/kg	Platinum-based chemotherapy
Naiyer 2020	Mystic	MN	III	721	Durvalumab 20 mg/kg	Platinum-based chemotherapy
Paz-Ares 2018	Keynote-407	United States	III	559	Pembrolizumab 200 mg + platinum-based chemotherapy	Platinum-based chemotherapy
Gandhi 2018	Keynote-189	MN	III	616	Pembrolizumab 200 mg + platinum-based chemotherapy	Platinum-based chemotherapy
Mok 2019	Keynote-042	MN	III	1,274	Pembrolizumab 200 mg	Platinum-based chemotherapy
Reck 2019	Keynote-024	MN	III	305	Pembrolizumab 200 mg	Platinum-based chemotherapy
Borghaei 2016	Keynote-021	United States, Taiwan	I/II	123	Pembrolizumab 200 mg + platinum-based Chemotherapy	Platinum-based chemotherapy
Rivero 2018	Impower 132	MN	Ⅲ	578	Atezolizumab 1200 mg + platinum-based Chemotherapy	Platinum-based chemotherapy
Robert 2018	Impower 131	MN	Ⅲ	1,021	Atezolizumab 1200 mg + platinum-based Chemotherapy	Platinum-based chemotherapy
West 2019	Impower 130	MN	Ⅲ	724	Atezolizumab 1200 mg + platinum-based Chemotherapy	Platinum-based chemotherapy
Spigel 2019	Impower 110	MN	III	572	Atezolizumab 1200 mg	Platinum-based chemotherapy
Hellmann 2018	Checkmate 227	MN	III	1,537	Nivolumab 3 mg/kg Nivolumab 3 mg/kg + Ipilimumab 1 mg/kg	Platinum-based chemotherapy

**TABLE 2:** Patient characteristics and extracted data for study end-points in the included randomised controlled trials.

Study ID	Treatment	Trail number	Dermatologic irAEs (pruritus and rash	Colitis	Endocrine irAEs (hypothyroidism and hyperthyroidism	Pneumonitis	Hepatitis
Checkmate 026	Nivolumab 3 mg/kg	267	92	3	20	23	60
	Platinum-based chemotherapy	263	48	0	7	17	36
Mystic	Durvalumab 20 mg/kg	369	107	1	40	26	33
	Platinum-based chemotherapy	352	88	0	4	10	37
Keynote-407	Pembrolizumab 200 mg + platinum-based chemotherapy	278	92	7	82	18	5
	Platinum-based chemotherapy	280	58	4	14	6	0
Keynote-189	Pembrolizumab 200 mg + platinum-basedchemotherapy	405	145	9	43	18	5
	Platinum-based chemotherapy	202	50	0	11	5	0
Keynote-042	Pembrolizumab 200 mg	636	107	7	116	53	9
	Platinum-based chemotherapy	615	44	2	13	3	0
Keynote-024	Pembrolizumab 200 mg	154	42	6	27	12	1
	Platinum-based chemotherapy	150	6	0	5	1	0
Keynote-021	Pembrolizumab 200 mg + platinum-based chemotherapy	59	22	2	14	3	1
	Platinum-based chemotherapy	62	11	0	4	0	0
Impower 132	Atezolizumab 1200 mg + platinum-based chemotherapy	291	75	5	23	16	3
	Platinum-based chemotherapy	274	60	0	6	6	1
Impower 131	Atezolizumab 1200 mg + platinum-based chemotherapy	334	74	6	34	23	4
	Platinum-based chemotherapy	334	39	0	4	5	0
Impower 130	Atezolizumab 1200 mg + platinum-based chemotherapy	473	119	4	53	28	3
	Platinum-based chemotherapy	232	28	0	1	14	0
Impower 110	Atezolizumab 1200 mg	286	NA	NA	NA	14	NA
	Platinum-based chemotherapy	263	NA	NA	NA	17	NA
Checkmate 227	Nivolumab 3 mg/kg	576	73	NA	NA	NA	NA
	Nivolumab 3 mg/kg + Ipilimumab 1 mg/kg	391	117	NA	NA	NA	NA
	Platinum-based chemotherapy	570	34	NA	NA	NA	NA

(Abbreviations: irAE, immune-related adverse event; MN,multinational; NA, not applicable.).

### Head-To-Head Comparisons for the Endpoints

**FIGURE 4:**
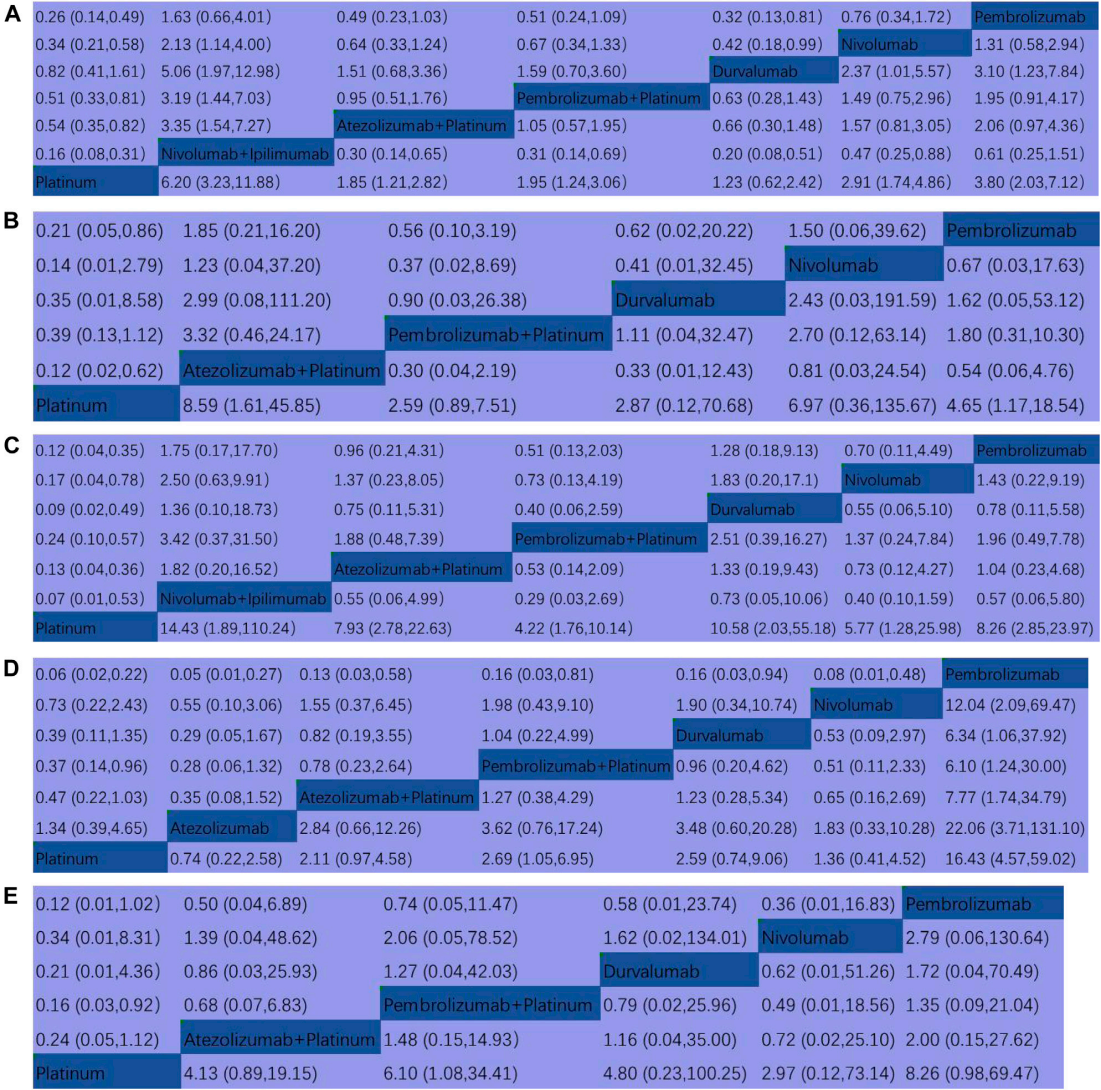
Pooled estimates of the network meta-analysis. **(A)** Multiple treatment comparison for dermatologic irAEs based on network consistency. **(B)** Multiple treatment comparison for colitis based on network consistency model. **(C)** Multiple treatment comparison for endocrine irAEs based on network consistency model. **(D)** Multiple treatment comparison for pneumonitis based on network consistency model. **(E)** Multiple treatment comparison for hepatitis based on network consistency model. (OR>1 means the treatment in top left is worse; Platinum = Platinum based chemotherapy).

In terms of dermatologic irAEs, platinum-based chemotherapy had the lowest rate compared to durvalumab (RR, 1.23, 95% CI, 0.62–2.42), atezolizumab + platinum (RR, 1.85, 95% CI, 1.21–2.82), pembrolizumab + platinum (RR, 1.95,95% CI, 1.24–3.06), nivolumab (RR, 2.91, 95%CI, 1.74–4.86), pembrolizumab (RR, 3.80, 95% CI, 2.03–7.12), nivolumab + ipilimumab (RR, 6.20, 95% CI, 3.23–11.88) (Figure 4A). In terms of colitis, platinum-based chemotherapy had the lowest rate compared to pembrolizumab + platinum (RR, 2.59, 95% CI, 0.89–7.51), durvalumab (RR, 2.87, 95% CI, 0.12–70.68), pembrolizumab (RR, 4.65, 95% CI, 1.17–18.54), nivolumab (RR, 6.97, 95% CI, 0.36–135.67), atezolizumab + platinum (RR, 8.59, 95% CI, 1.61–45.85), (Figure 4B). For endocrine irAEs, platinum-based chemotherapy had the lowest rate compared to pembrolizumab + platinum (RR, 4.22, 95% CI, 1.76–10.14), nivolumab (RR, 5.77, 95% CI, 1.28–25.98), Atezolizumab + platinum (RR, 7.93, 95% CI, 2.78–22.63), pembrolizumab (RR, 8.26, 95% CI, 2.85–23.97), durvalumab (RR, 10.58, 95% CI, 2.03–55.18), and nivolumab + ipilimumab (RR, 14.43, 95% CI, 1.89–110.2) (Figure 4C). For pneumonitis, atezolizumab had the lowest rate compared to platinum-based chemotherapy (RR,1.34, 95% CI, 0.39–4.65), nivolumab (RR, 1.83, 95% CI, 0.33–10.28), atezolizumab + platinum (RR, 2.84, 95% CI, 0.66–12.26), durvalumab (RR, 3.48, 95% CI, 0.60–20.28), pembrolizumab + platinum (RR, 3.62, 95% CI, 0.76–17.24), and pembrolizumab (RR, 22.06, 95% CI, 3.71–131.10) (Figure 4D). For hepatitis, pembrolizumab + platinum had the lowest rate compared to nivolumab (RR, 2.97, 95% CI, 0.12–73.14), atezolizumab + platinum (RR, 4.13, 95% CI, 0.89–19.15), durvalumab (RR, 4.80, 95% CI, 0.23–100.25), pembrolizumab + platinum (RR, 6.10, 95% CI, 1.08–34.41), pembrolizumab (RR, 8.26, 95% CI, 0.98,69.47) (Figure 4E).

### Determining the Ranking

**FIGURE 5:**
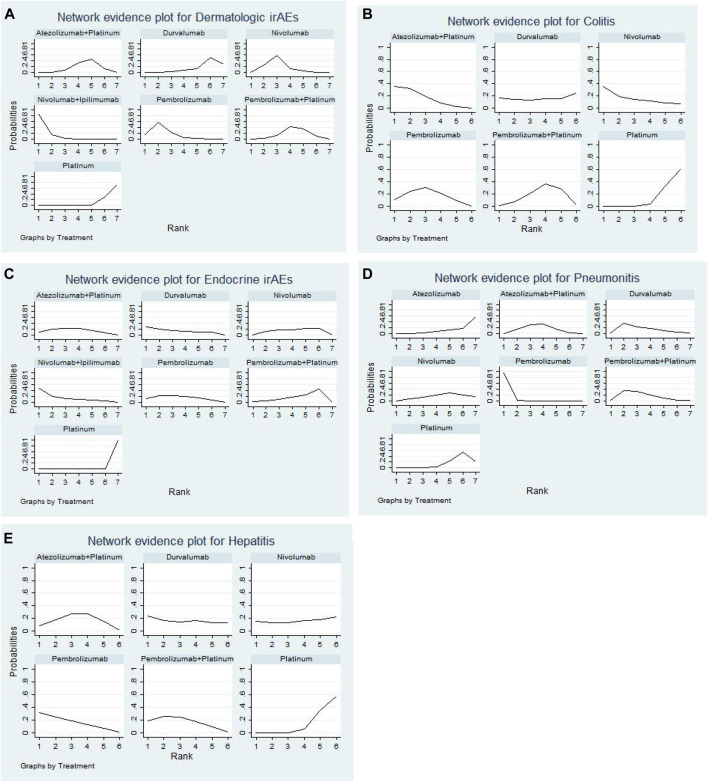
Sequence diagram of the network meta-analysis. **(A)** Multiple treatment comparison for dermatologic irAEs based on SUCRA. **(B)** Multiple treatment comparison for colitis based on SUCRA. **(C)** Multiple treatment comparison for endocrine irAEs based on SUCRA. **(D)** Multiple treatment comparison for pneumonitis based on SUCRA. **(E)** Multiple treatment comparison for hepatitis based on SUCRA. (Platinum = Platinum based chemotherapy).

We ranked the probabilities of immune-related adverse events for all treatments by estimating the SUCRA value. A higher SUCRA value indicated a higher probability of irAEs and a poorer treatment regimen. For dermatologic irAEs, the corresponding ranking of incidences of the seven groups from high to low was: nivolumab + ipilimumab (97.4%), pembrolizumab (80.1%), nivolumab (67.1%), pembrolizumab + platinum (43.3%), atezolizumab + platinum (39.9%), durvalumab (17.5%), platinum-based chemotherapy (4.7%) (Figure 5A, Supplementary figure S1A). For colitis, the corresponding ranking of incidences of the six groups from high to low was: atezolizumab + platinum (77.1%), nivolumab (67.3%), pembrolizumab (60.5%), durvalumab (45.2%), pembrolizumab + platinum (41.4%), platinum-based chemotherapy (8.5%) (Figure 5B, Supplementary figure S1B). For endocrine irAEs, the corresponding ranking of incidences of the seven groups from high to low was: nivolumab + ipilimumab (79.1%), durvalumab (69.1%), pembrolizumab (61.9%), atezolizumab + platinum (60.4%),nivolumab (45.7%), pembrolizumab + platinum (33.5%), platinum-based chemotherapy (0.3%) (Figure 5C, Supplementary figure S1C). For pneumonitis, the corresponding ranking of incidences of the seven groups from high to low was: pembrolizumab (99.3%), pembrolizumab + platinum (65.1%), durvalumab (62.2%), atezolizumab + platinum (56%), nivolumab (35.9%), platinum-based chemotherapy (18.1%),atezolizumab (13.3%) (Figure 5D, Supplementary figure S1D). For hepatitis, the corresponding ranking of incidences of the six groups from high to low was: pembrolizumab (71.2%), pembrolizumab + platinum (64.3%), durvalumab (56.4%), atezolizumab + platinum (53.8%), nivolumab (44.5%), platinum-based chemotherapy (9.8%) (Figure 5E, Supplementary figure S1E).

### Convergence, Heterogeneity and Publication Bias

**FIGURE 6:**
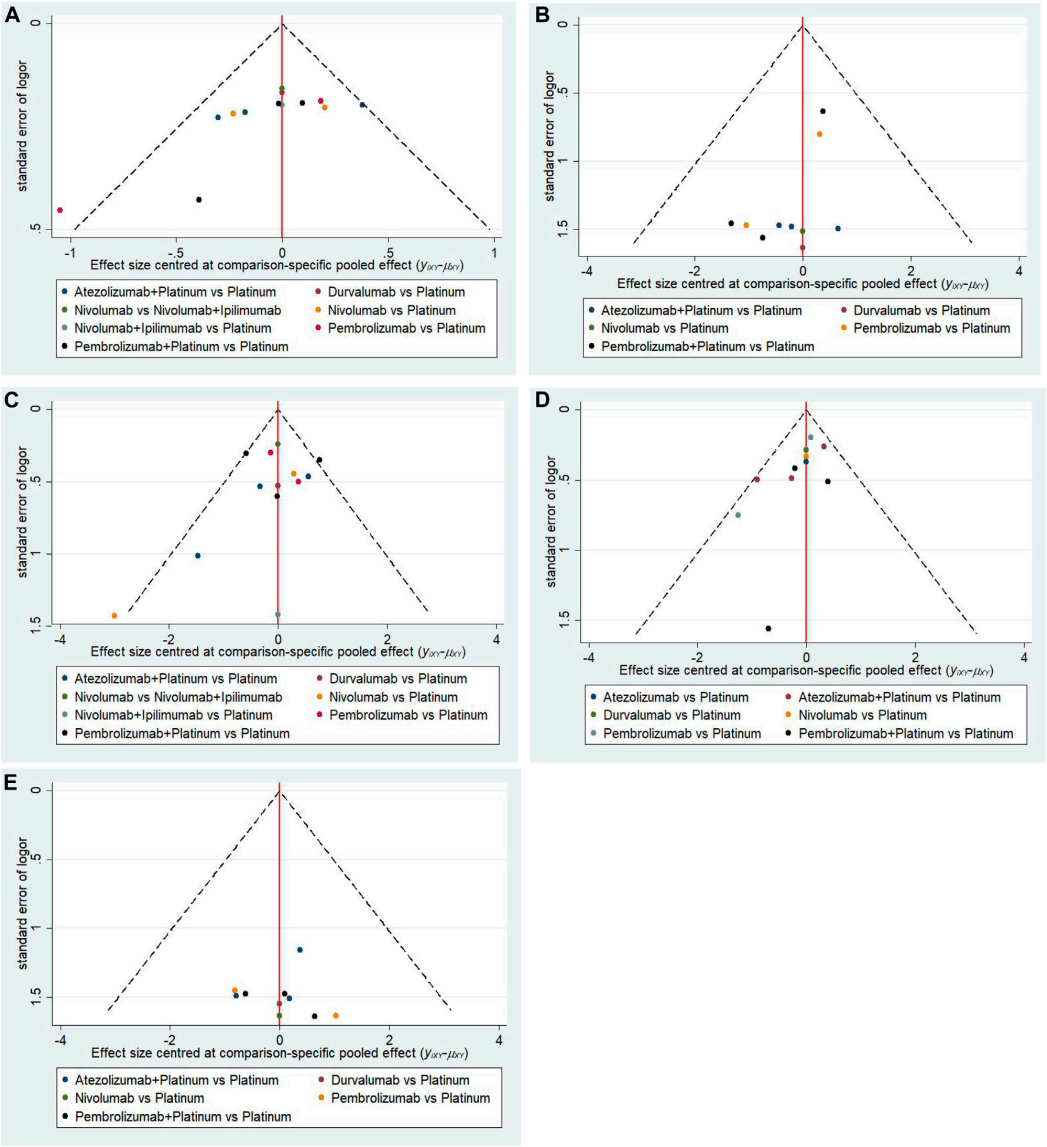
Funnel plot of **(A)** dermatologic irAEs, **(B)** colitis, **(C)** endocrine irAEs, **(D)** pneumonitis and **(E)**hepatitis in the network meta-analysis.(Platinum = Platinum based chemotherapy).

As shown in Supplementary figure S2, all comparisons of different irAEs all suggested that the contraction factor in Brookse-Gelmane-Rubin diagnostic plots were equal to the predefined cut-off value 1, suggesting that the study model had good convergence. In this network meta-analysis the comparisons showed low, medium or high heterogeneity (Supplementary figure S3), then the random effects model was selected. The funnel plots were all symmetrically distributed, suggesting no publication bias (Figure 6).

### Risk-Of-Bias Assessment and Sensitivity Analysis

As shown in Figure 1, 9 included RCTs (Corey et al., 2016; Reck et al., 2016; Carbone et al., 2017; Barlesi et al., 2018; Hellmann et al., 2019; West et al., 2019; Jotte et al., 2020; Naiyer et al., 2020; Roy et al., 2020) were regarded as high risk on blinding of participants and personnel and 5 included RCTs (Gandhi et al., 2018; Paz-Ares et al., 2018; Tony et al., 2019; Jotte et al., 2020; Naiyer et al., 2020) were considered high risk on incomplete outcome data. Other domains indicated unclear risk or low risk. The inconsistency test conducted that *p* values>0.05 indicated there was significant consistency between direct and indirect results of all comparisons, and then the sensitivity analysis indicated stable results (Supplementary figure S4A-E).

## Discussion

In recent years, a number of RCTs had successively evaluated ICI + chemotherapy, ICI alone, dual ICIs combination versus chemotherapy in terms of efficacy and safety in the first-line treatment of advanced NSCLC (Corey et al., 2016; Reck et al., 2016; Carbone et al., 2017; Barlesi et al., 2018; Gandhi et al., 2018; Paz-Ares et al., 2018; Hellmann et al., 2019; Tony et al., 2019; West et al., 2019; Jotte et al., 2020; Naiyer et al., 2020; Roy et al., 2020). These RCTs suggested that the efficacy and treatment-related side effects of ICI + chemotherapy, ICI alone or dual ICIs were better than chemotherapy alone. Because of this, ICIs including nivolumab (Kazandjian et al., 2016), pembrolizumab (Pai-Scherf et al., 2017) and atezolizumab (Weinstock et al., 2017) were approved for NSCLC by FDA and had became an important part of treatment options in advanced NSCLC (Antonia et al., 2017). However with the widespread use of ICIs, the irAEs showed a tendency to be more prevalent in ICIs than in chemotherapy. IrAEs represented an entirely new toxicity spectrum of ICIs, and it could affect any tissue or organ, if mishandled, could reduce the survival benefit of antitumor efficacy, which might be related to the mechanism by which ICI alters the balance of immune cells in the body, inducing damage to some organ systems. The incidence and severity of irAEs were usually related to the drug type, tumor type, irAEs history and other immune-related medical history (Chen et al., 2015), and different ICIs had different immune mechanisms, and even belonging to the same mechanism had different tolerability to different irAEs. However which treatment regime could be better tolerated was worth studying.

Here, we indirectly compared the probability of irAEs caused by ICI + platinum, ICI alone, dual ICIs combination for advanced NSCLC through NMA. Data on irAEs from published studies and Clinicaltrials.gov from 2015 to 2020 were collected. Finally, 12 head-to-head phase II and III RCTs (Corey et al., 2016; Reck et al., 2016; Carbone et al., 2017; Barlesi et al., 2018; Gandhi et al., 2018; Paz-Ares et al., 2018; Hellmann et al., 2019; Tony et al., 2019; West et al., 2019; Jotte et al., 2020; Naiyer et al., 2020; Roy et al., 2020)including 8,453 patients with advanced NSCLC were included in this network meta-analysis. Notably, this NMA concluded that for dermatologic irAEs, colitis, endocrine irAEs, pneumonitis and hepatitis, ICI-based therapy showed a higher incidence of irAEs than platinum-based chemotherapy. Qiang Su (Su et al., 2019) concluded that compared with chemotherapy, PD-1/PD-L1 inhibitors showed significant increase in grade 1–5 and grade 3–5 pneumonitis, which was consistent with the results of ours. And for dermatologic irAEs, colitis, endocrine irAEs, pneumonitis and hepatitis, pembrolizumab + platinum showed a higher incidence of irAEs than pembrolizumab, which was consistent with Slater et al.'s (Slater et al., 2002) conclusion that chemotherapy may produce immunosuppression. Interestingly, only dermatologic and endocrine irAEs involved nivolumab + ipilimumab and we concluded that nivolumab + ipilimumab showed a higher incidence of dermatologic and endocrine irAEs than ICI + platinum and ICI alone and the explanation for this might be that the combination of the dual ICIs increased the imbalance of immune cells and led to more irAEs. And we found something new that except for chemotherapy, durvalumab for dermatologic irAEs, pembrolizumab + platinum for colitis, pembrolizumab + platinum for endocrine irAEs, atezolizumab for pneumonitis and nivolumab for hepatitis had the least incidence of irAEs, which were different from the conclusion of Cheng Xu et al. (Xu et al., 2018) and Xinru Chen et al. (Chen et al., 2020). Cheng Xu et al. (Xu et al., 2018) compared the occurrences of irAEs with different immunotherapy regimens for all cancers in 2018, and they compared the efficacy of ICI monotherapy, ICI monotherapy plus chemotherapy, and chemotherapy in lung cancer and concluded that atezolizumab had the best overall safety, while nivolumab had the best overall safety in the treatment of lung cancer with a combined approach. Xinru Chen et al. (Chen et al., 2020) investigated different immunotherapy regimens for immune-related pneumonia (IRP), and they concluded that ICIs increased the risk of IRP compared to chemotherapy and that ICI + chemotherapy was associated with a lower risk of IRP than dual ICIs combination and ICI monotherapy. However the reason why my results were different from theirs might be: firstly, compared to previous studies, we only included patients with advanced NSCLC and excluded other cancer types in this NMA, which was relatively specific, and all data were up to date. Secondly, the studies of us that only platinum-based chemotherapy was included, and those involving docetaxel were excluded. Thirdly, instead of integrating different drugs with similar mechanisms into the same arm, we analyzed each of the treatment regimens separately.

The current analysis has several strengths. Firstly, to our knowledge, this NMA analyzed the probability of irAEs caused by different treatment regimens for advanced NSCLC more specifically. Secondly, some conclusions this study drew were innovative compared to previous studies, which will have novel significance for guiding clinical treatment and subsequent studies. Thirdly, all of the articles included in this NMA were RCTs and the Cochrane risk bias assessment tool were used to examine the quality of included studies and to ensure their inherent authenticity by assessing the potential risk of bias in various aspects of RCT design, implementation, and outcome evaluation. Fourthly, in this NMA, in order to detect the heterogeneity of the included literature and data, we used heterogeneity test, and in order to detect publication bias, we used funnel plots, and the results showed that no significant publication bias was found. Fifthly, we conducted sensitivity analysis on all studies to investigate inconsistencies between direct and indirect comparisons by inconsistency test, and the results all suggested that *p*>0.05, indicating that our research results are stable. All of these suggested that our study is quite reliable.

Nonetheless, there were a few unavoidable limitations to our article. Firstly, in all of the studies of irAEs included, almost all comparisons showed low heterogeneity except for some comparisons. Secondly, although we found that the rating systems and terminology used in the reports were consistent and compatible, the diagnosis of each irAE was based solely on the experience of each clinician, rather than on a centralized review, which can lead to bias in irAE evaluation. Thirdly, some irAEs were delayed diseases, and clinicians could not have observed the symptoms of patients within the clinical timeframe, leading to the loss of clinical data, which would bring some potential heterogeneity to this study. Fourthly, the median follow-up time for each RCT was different, which would increase the frequency of irAEs associated with immunotherapy and increase the confounding factors for these events. Fifthly, although we included the latest RCT data that could be retrieved, some studies had a small sample size, which might be the reason why some comparisons showed moderate or high heterogeneity.

Despite there are some limitations in our study, we still have some prospects for the prediction and treatment of irAEs. According to the reports (Wang et al., 2019), most irAEs have been reported to be mild, such as cutaneous and endocrine, but death from moderate, severe or life-threatening irAEs have been reported in 1–2% of patients, such as pneumonia and colitis and T. W. Chen et al. (Chen et al., 2015) suggest that seventeen percent of studies over stated the safety of the experimental regimen, and although glucocorticoids and steroids (Weber et al., 2009) can be used for irAES, some patients still die because of late diagnosis, which emphasize the importance of determining the predictors of irAEs and early management and prevention. Studies have suggest that after treatment of ICI peripheral blood eosinophil is associated with irAEs (Schindler, 2014). Circulating IL-17 (Callahan, 2011), expression of CD177 and CEACAM1 (Shahabi et al., 2013), and neutrophil infiltration in the lamina propria of the colon (Berman et al., 2010) is suggested to be associated with gastrointestinal toxicity, however the occurrence mechanism of other irAEs is not clear, and at present, the evaluation system of irAEs is not perfect. In the future, more effective predictors of irAEs will be needed and with the extensive application of ICIs treatment and the development of related trials, people will have a fuller understanding of the mechanism of irAEs, and the management measures will be more standardized.

## Conclusion

This systematic review and NMA suggests that, in addition to platinum-based chemotherapy, durvalumab for dermatologic and liver irAEs, pembrolizumab for gastrointestinal irAEs, pembrolizumab + platinum for endocrine irAEs, and atezolizumab for pneumonitis may be associated with lower rates of irAEs than other immune-based regimens. Nivolumab + ipilimumab for dermatologic and endocrine irAEs, atezolizumab + platinum for colitis, and pembrolizumab for pneumonitis and hepatitis may be associated with higher rates of irAEs. These conclusions will be helpful for clinical management, early prediction, early detection and early treatment. However a large number of multicenter clinical trials and studies are still needed to balance the therapeutic efficacy and the occurrence of irAEs of ICIs to maximize patient benefit.

## Data Availability

The original contributions presented in the study are included in the article/Supplementary Material, further inquiries can be directed to the corresponding author.

## Abbreviations

ICI, immune checkpoint inhibitor; ICIs, immune checkpoint inhibitors; irAEs, immune-related adverse events; NSCLC, non-small cell lung cancer; RCTs, randomized clinical trials; RRs, risk ratios; 95%CI, 95% confidence interval; NMA, network meta-analysis; CTLA4, cytotoxic T lymphocyte-associated antigen-4; PD-1, programmed cell death receptor 1; PD-L1, programmed cell death ligand-1; PD-L2, programmed cell death ligand-2; PRISMA, preferred reporting items for systematic reviews and meta-analysis; MCMC, Markov chain Monte Carlo; SUCRA, surface under the cumulative ranking curve.
