# Supplementary material for: Immune-Related Adverse Events Associated With Immune Checkpoint Inhibitors for Advanced Non-small Cell Lung Cancer: A Network Meta-Analysis of Randomized Clinical Trials
Source: Front Pharmacol. 2021 Oct 25;12:686876. doi: 10.3389/fphar.2021.686876 (PMC8574003; doi:10.3389/fphar.2021.686876)

**Supplementary Materials:**

**Figure1**

**A**

**
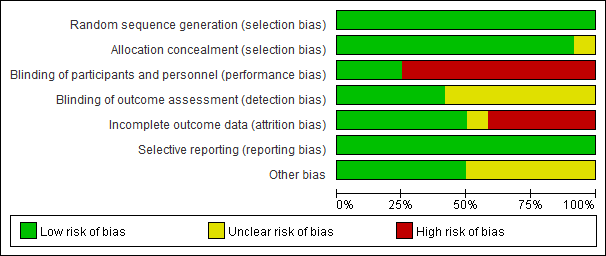
**

**1B**

**
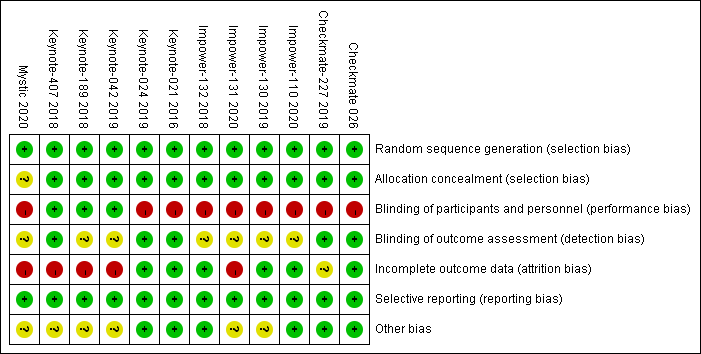
**

**Figure2**

**
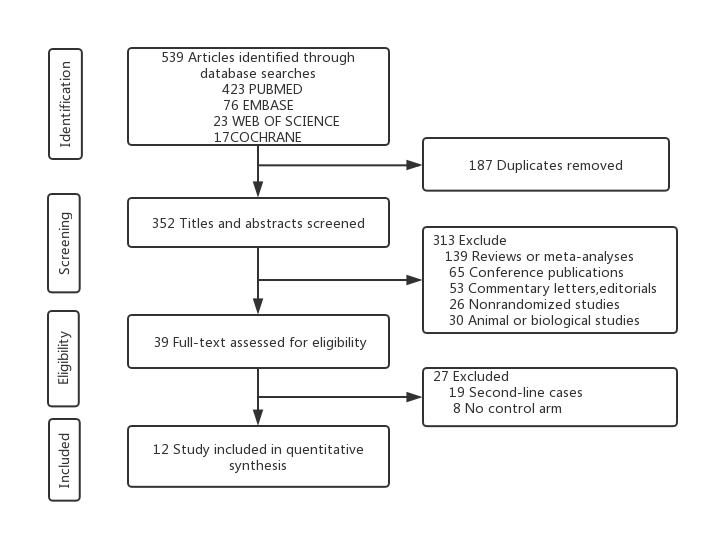
**

**Figure 3**

**3A**

**
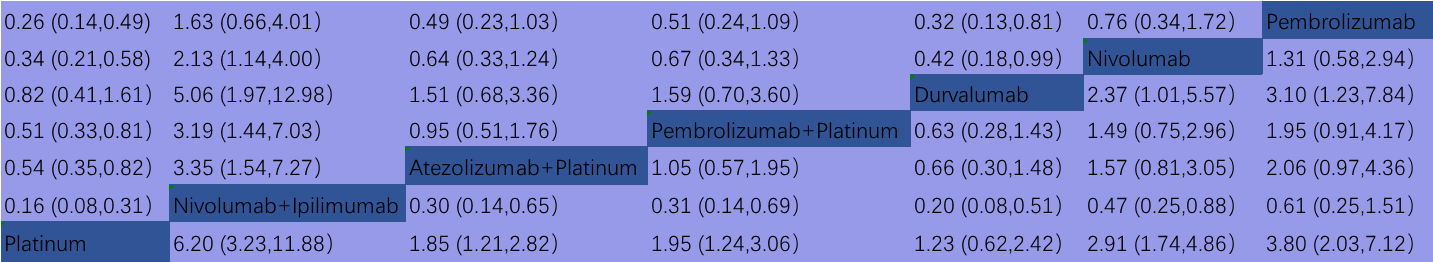
**

**3B**

**
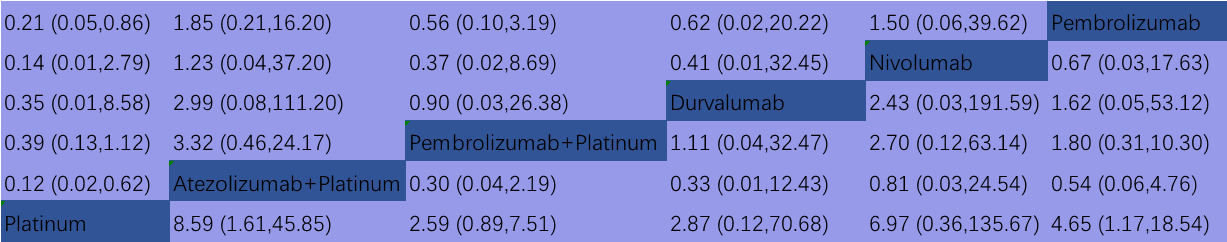
**

**3C**

**
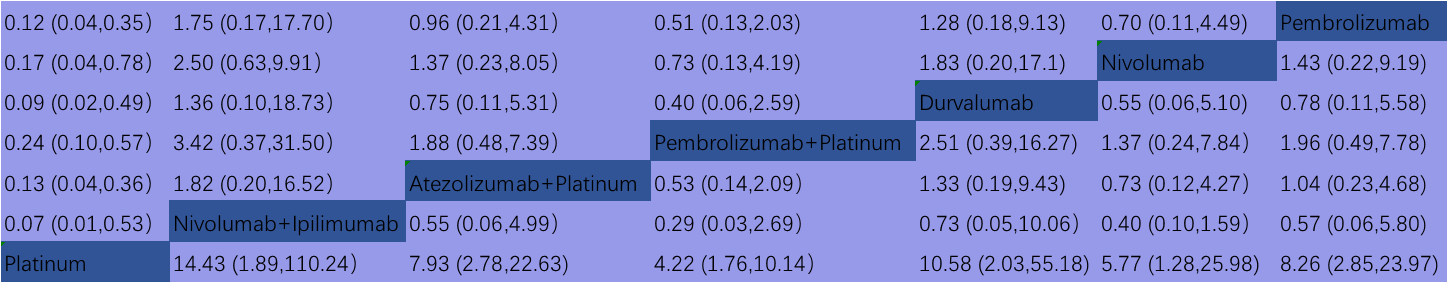
**

**3D**

**
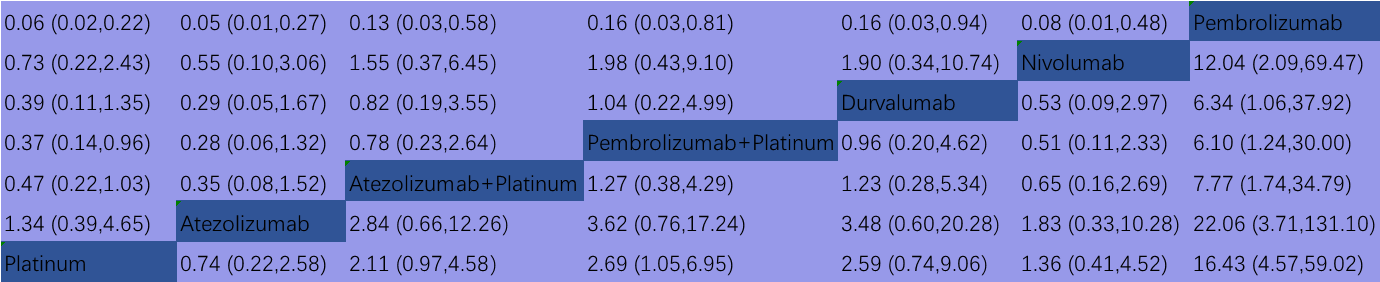
**

**3E
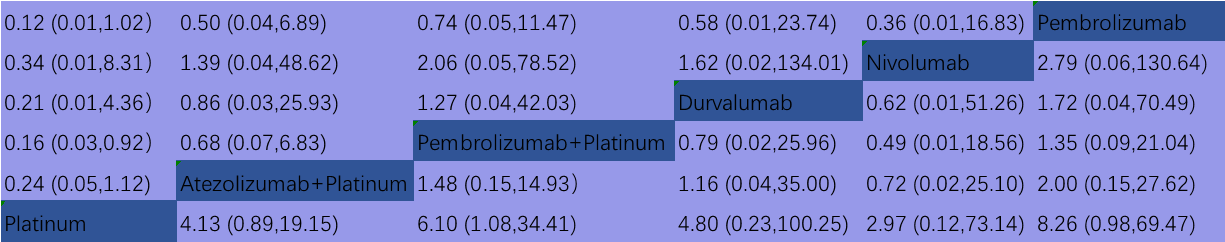
**

**Figure 4**

**4A**

**
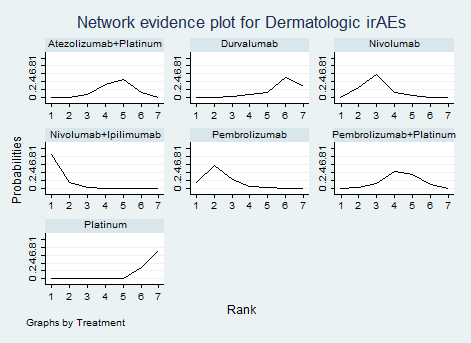
**

**4B**

**
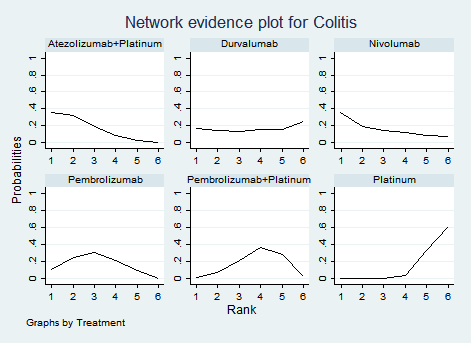
**

**4C**

**
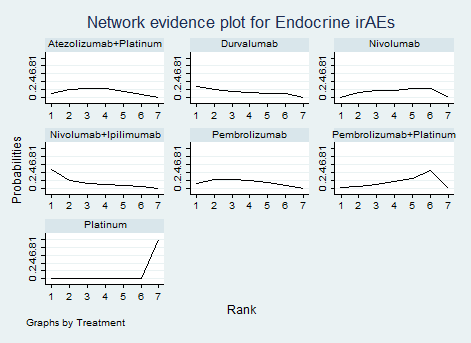
**

**4D**

**
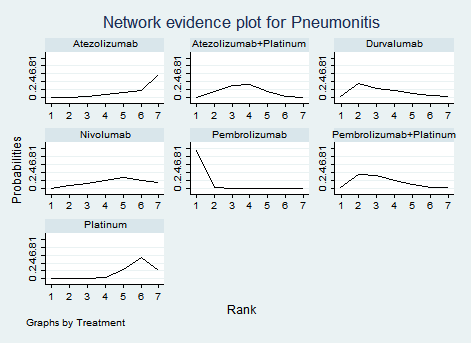
**

**4E**

**
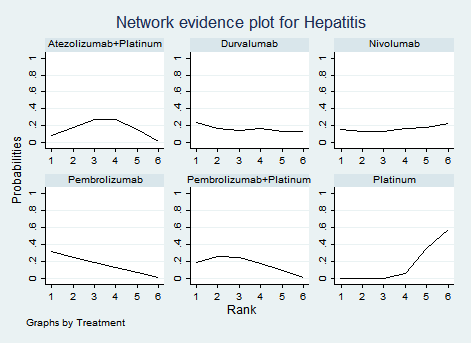
**

**Figure 5**

**5A**


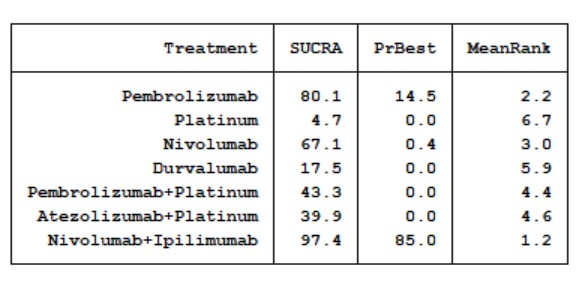


**5B**

**
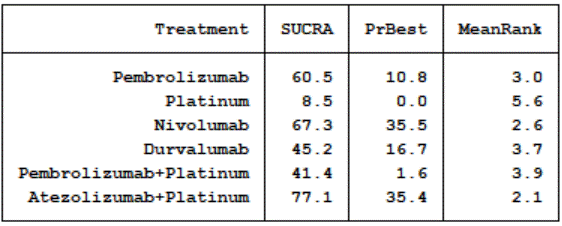
**

**5C**
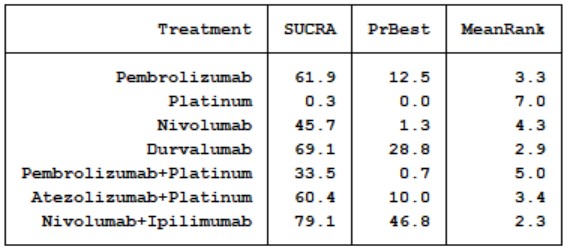


**5D**

**
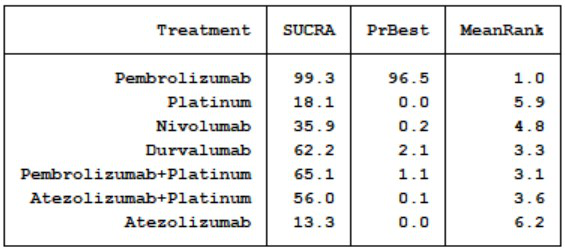
**

**5E**

**
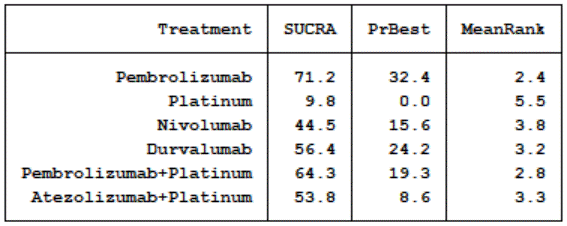
**

**Figure 6**

**6A**


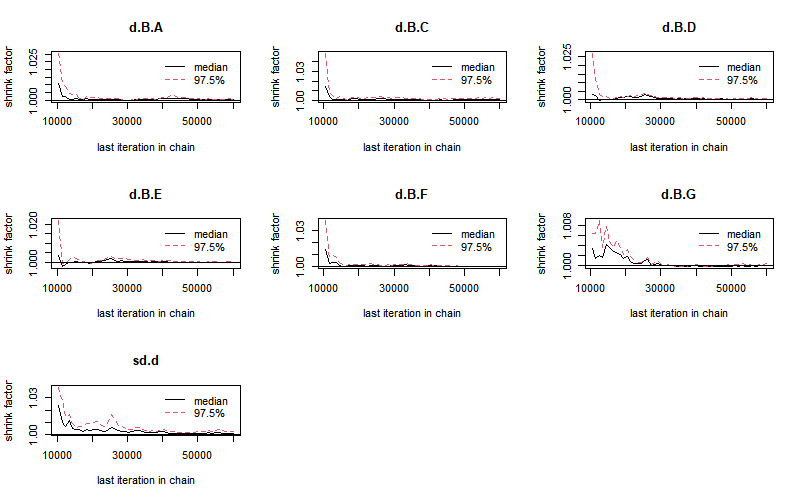


**6B**
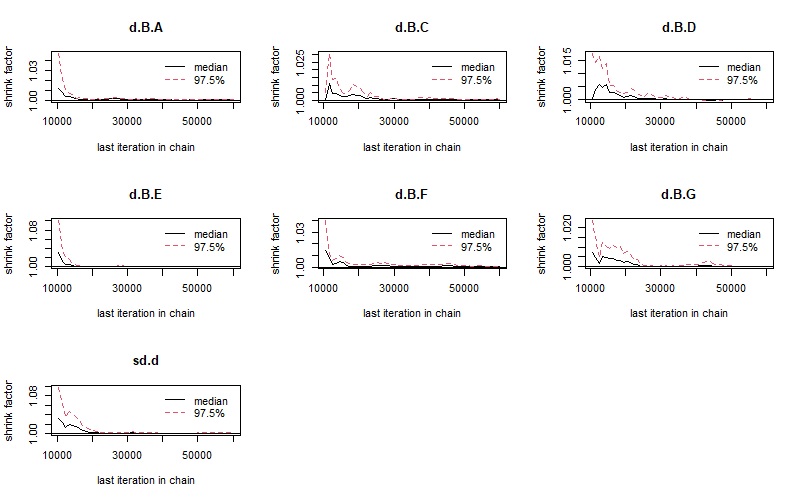


**6C**


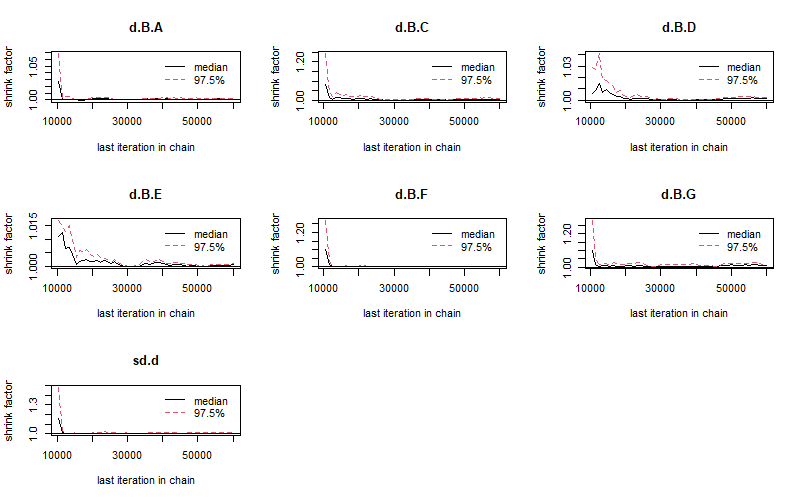


**6D**


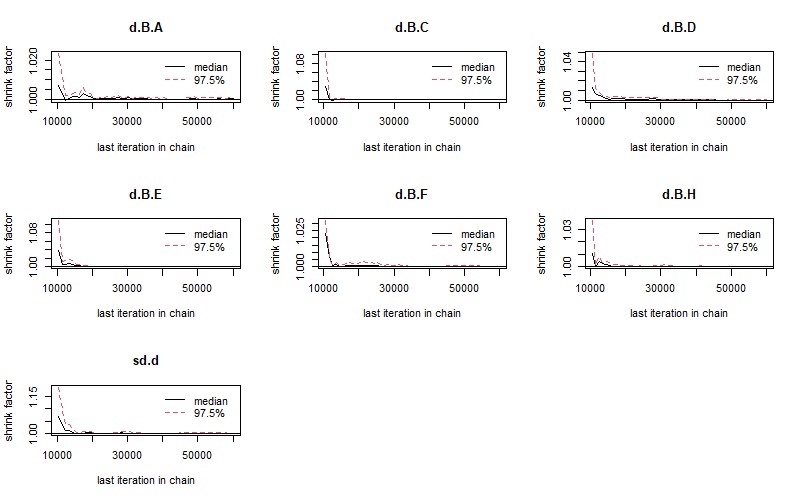


**6E**


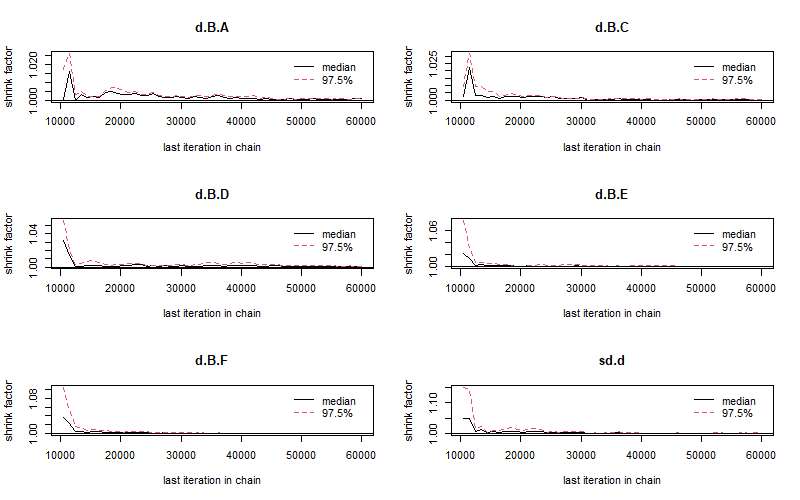


**Figure7**

**7A**


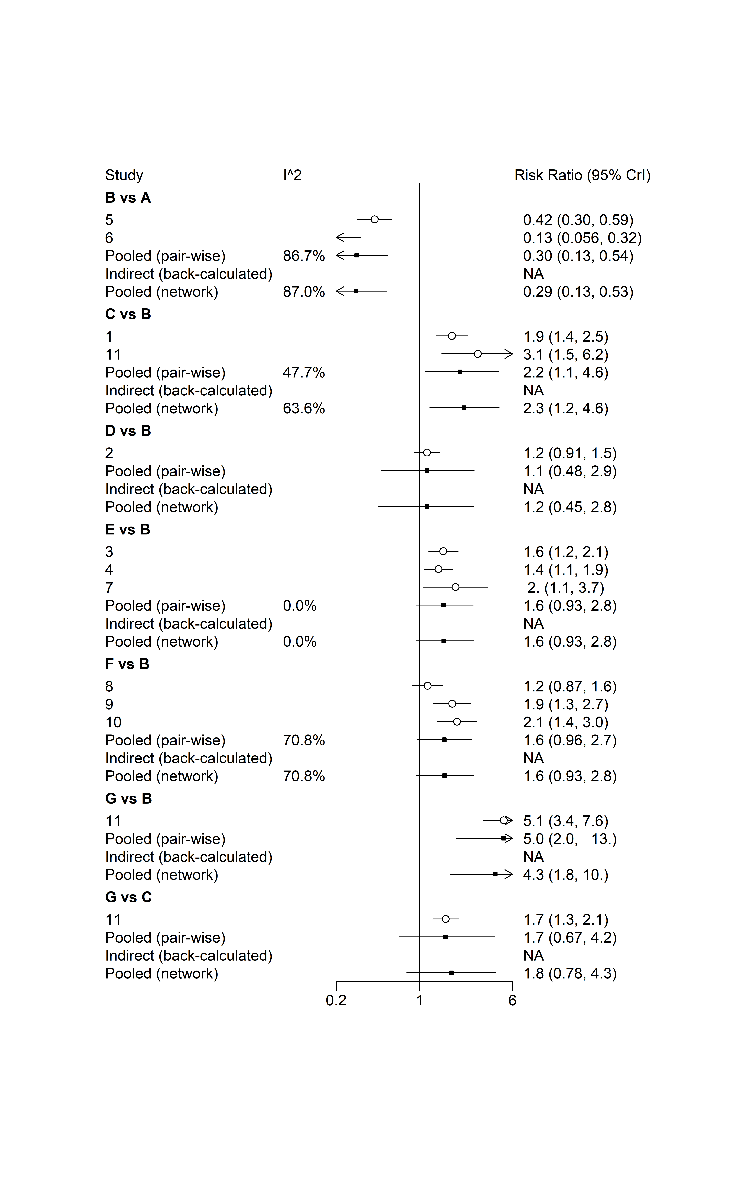


**7B**


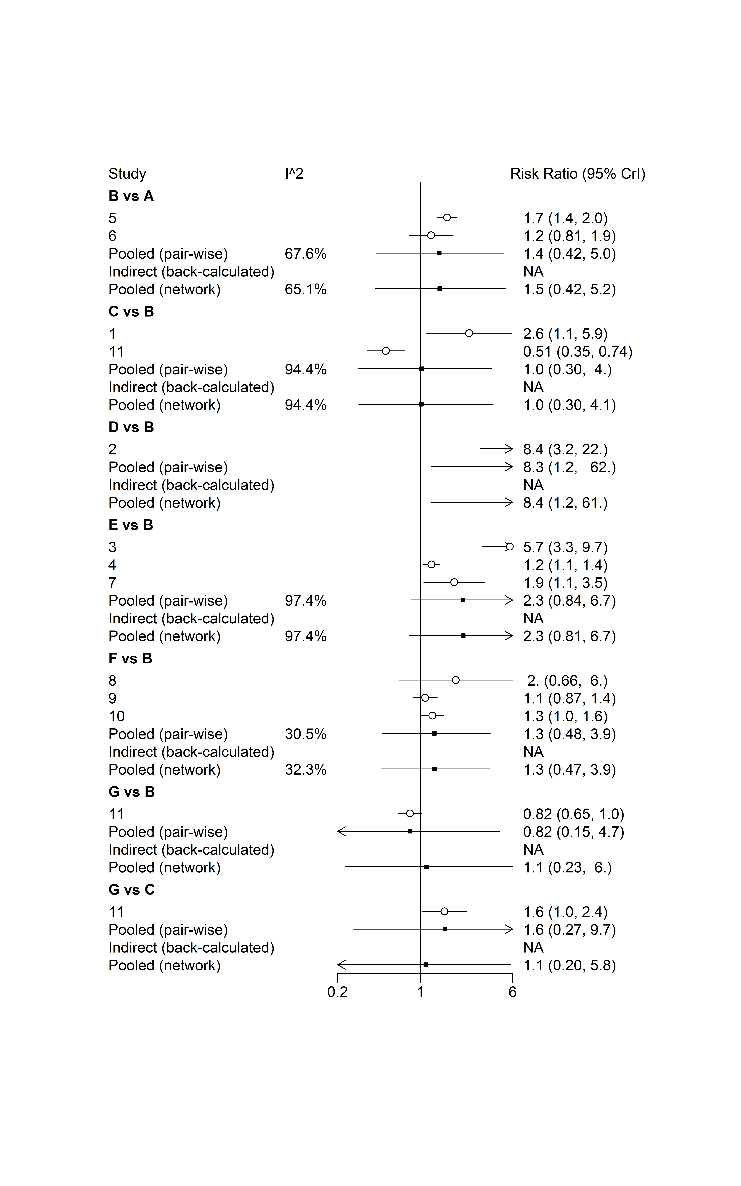


**7C**


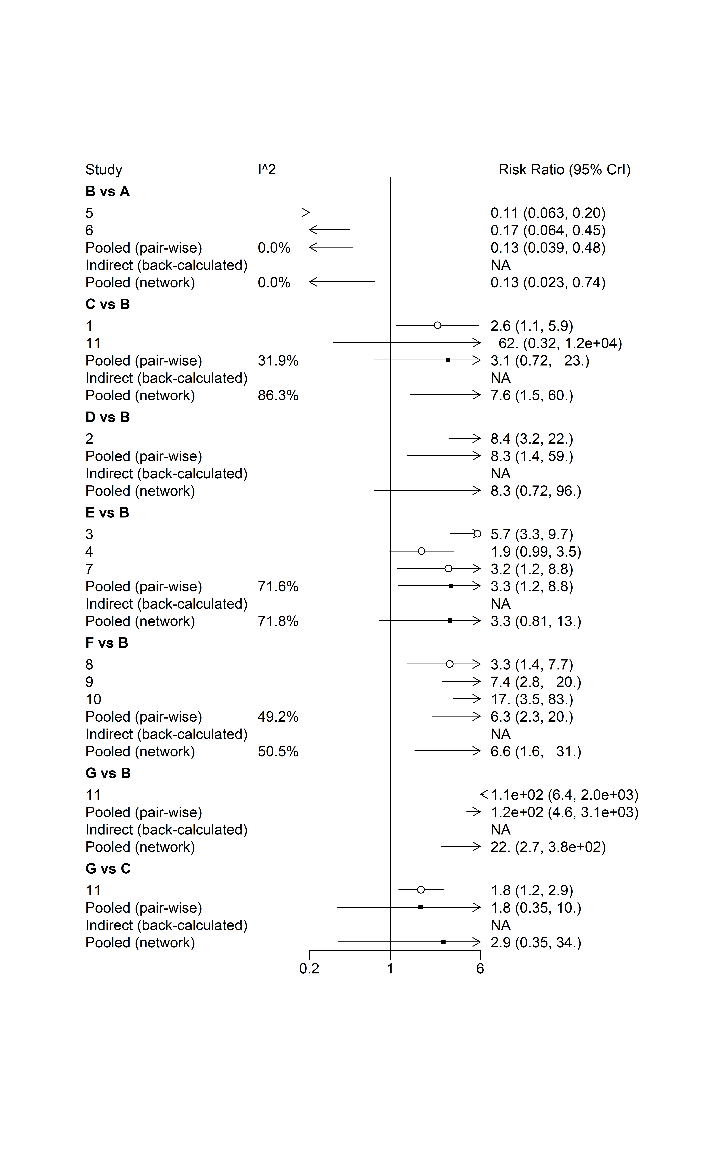


**7D**


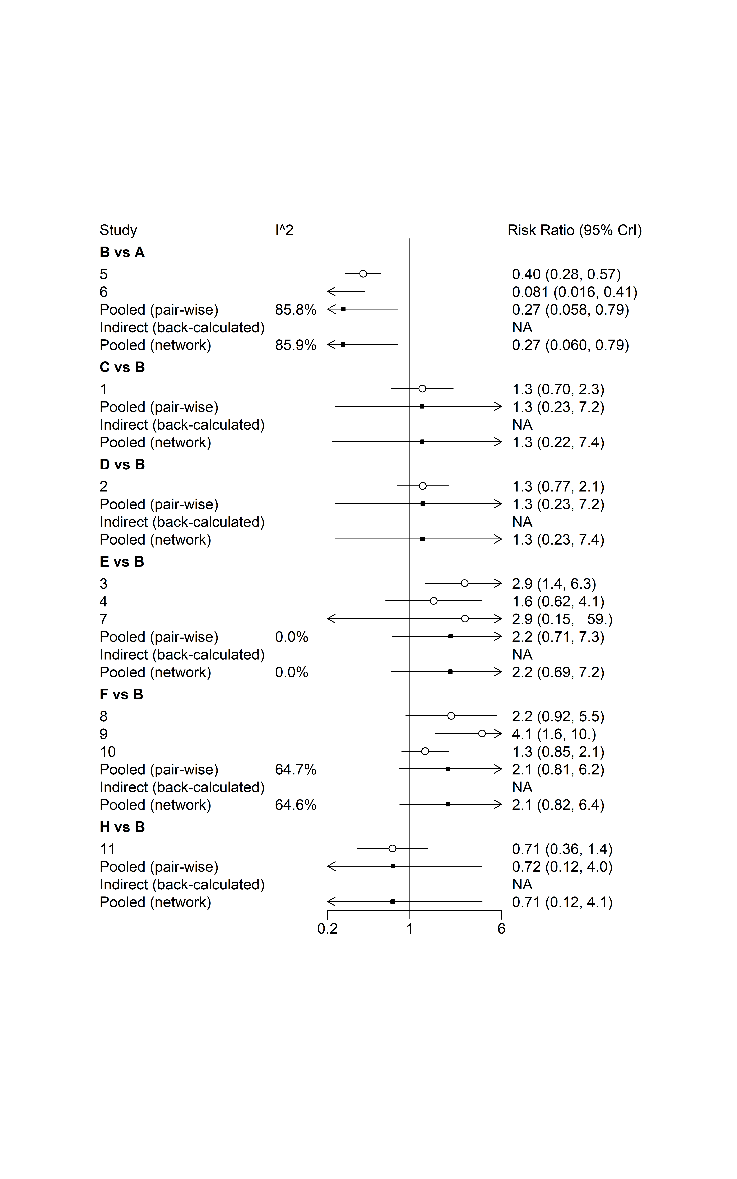


**7E**


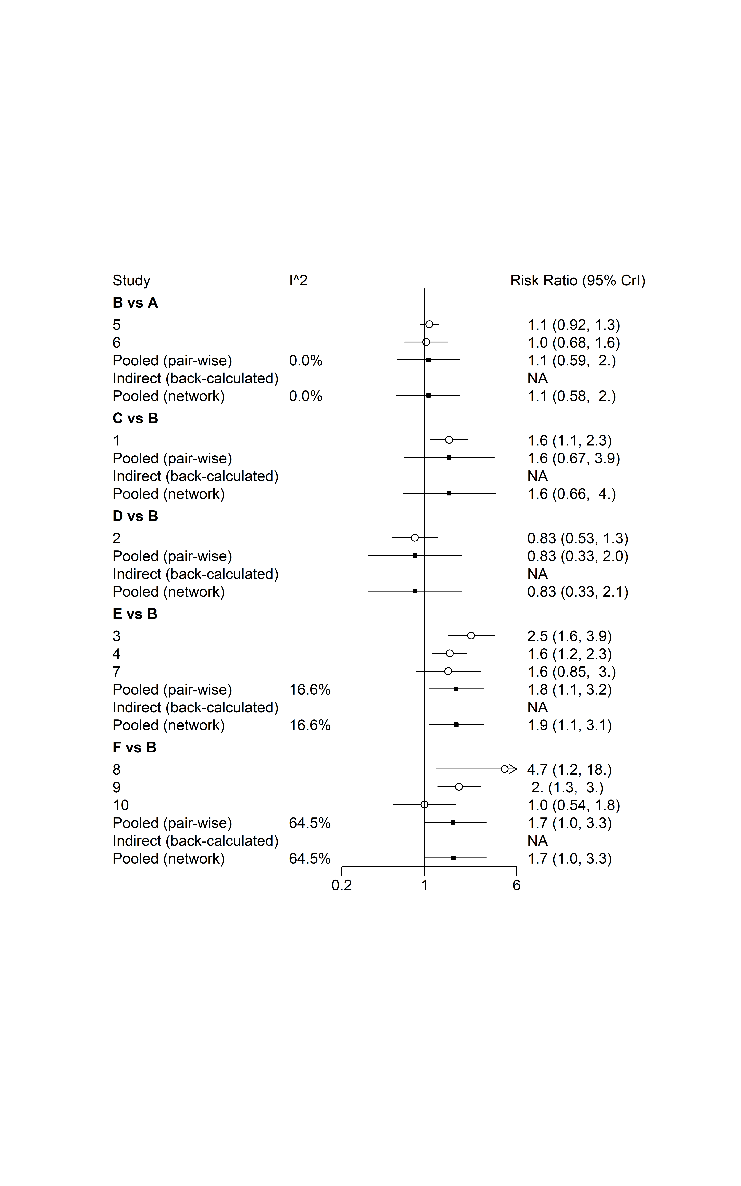


**Figure8**

**8A**

**
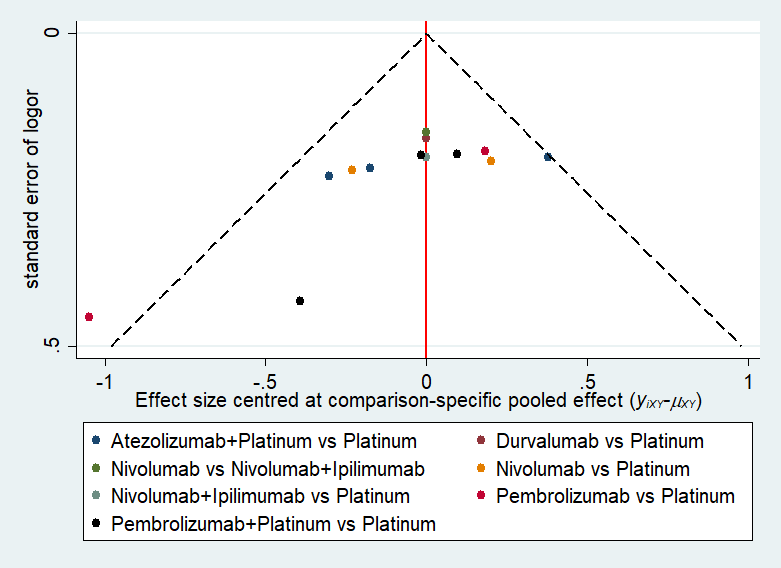
**

**8B**

**
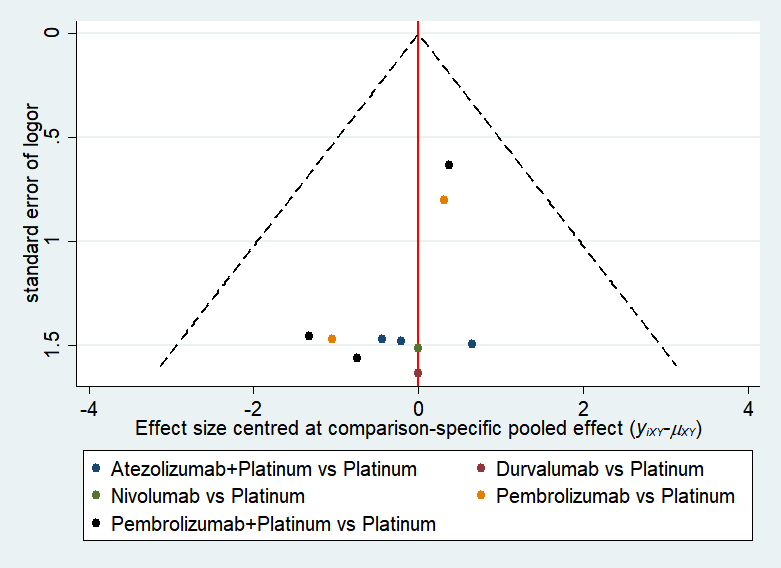
**

**8C**

**
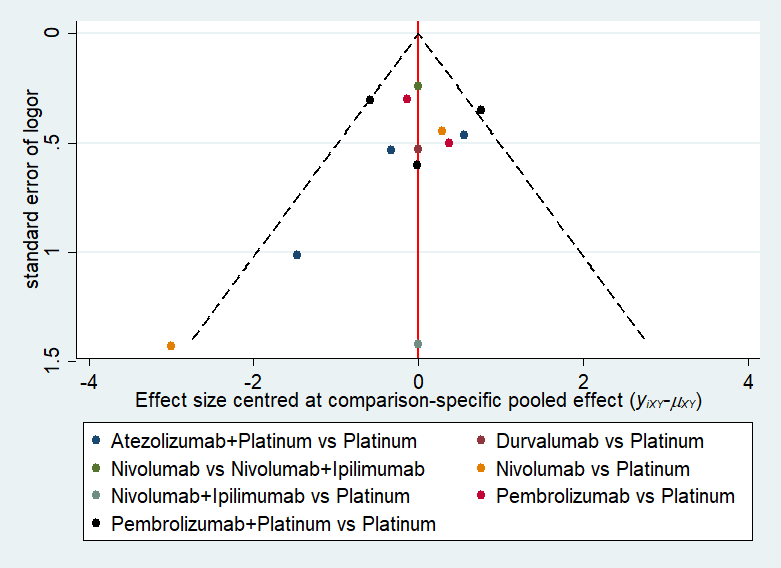
**

**8D**

**
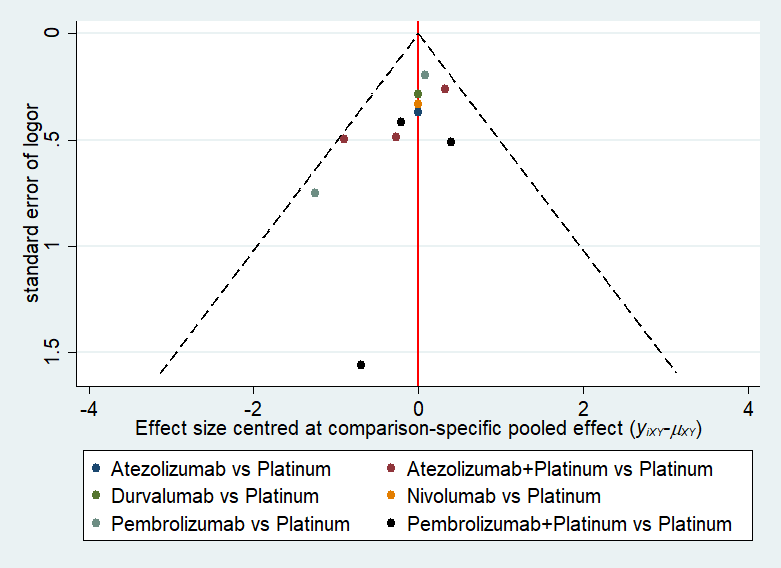
**

**8E**

**
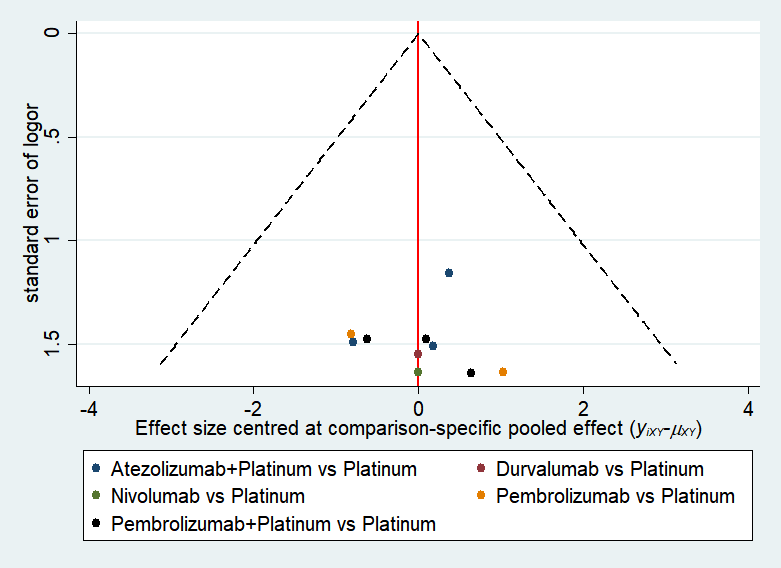
**

**Figure 9**

**9A**
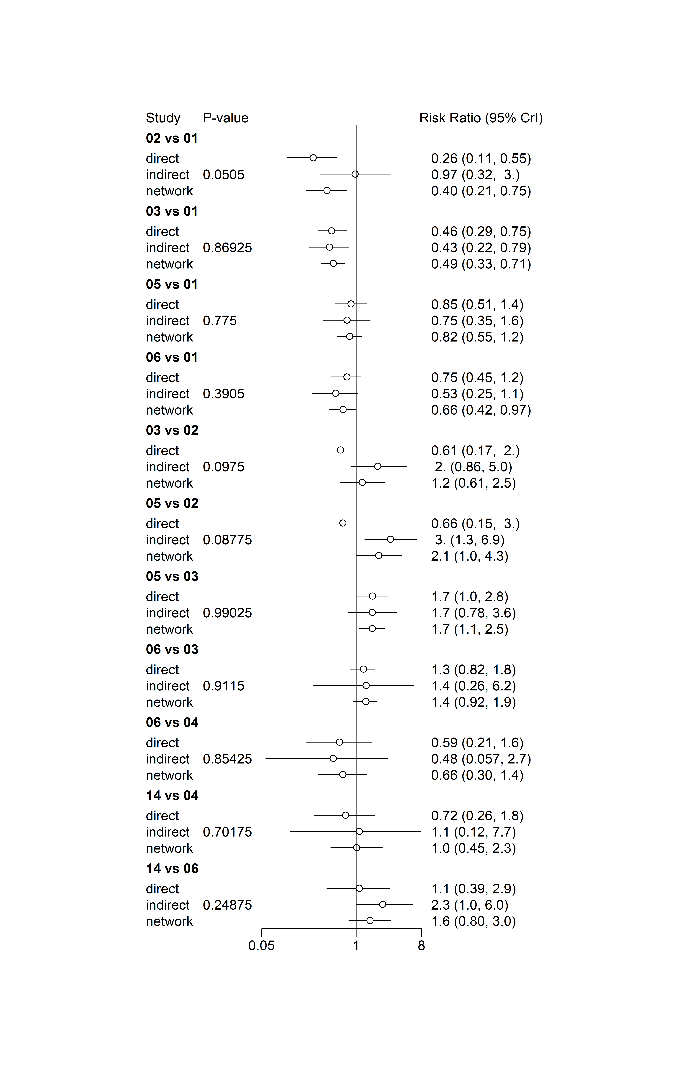


**9B**

**
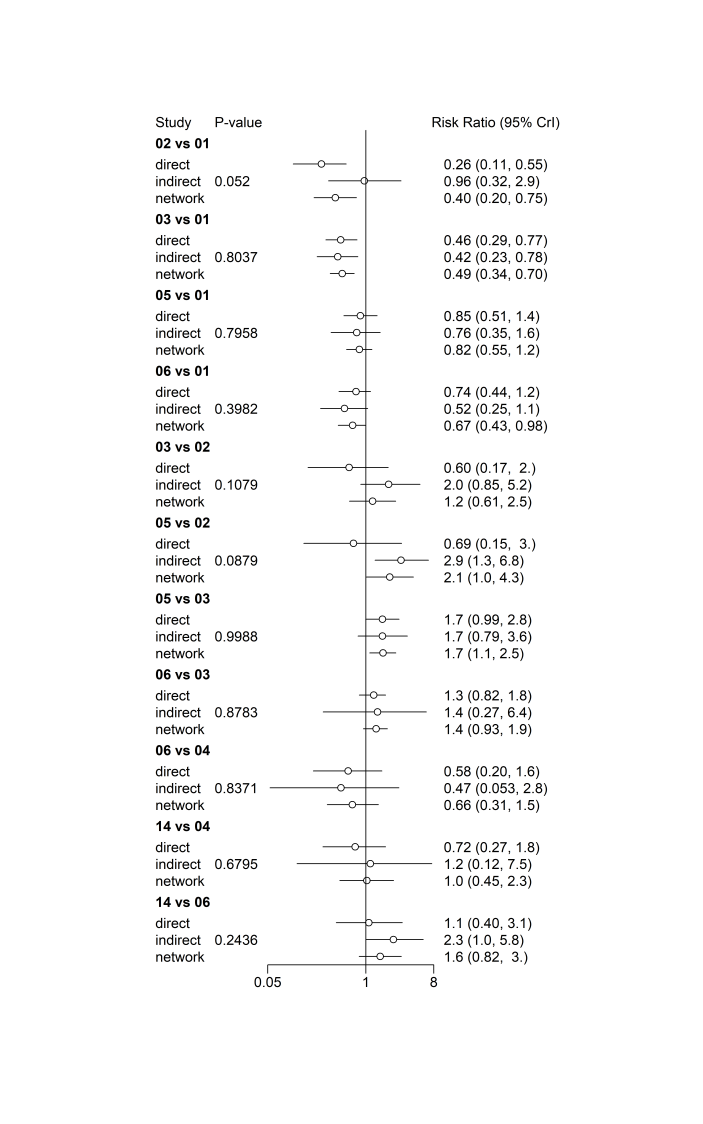
**

**9C**


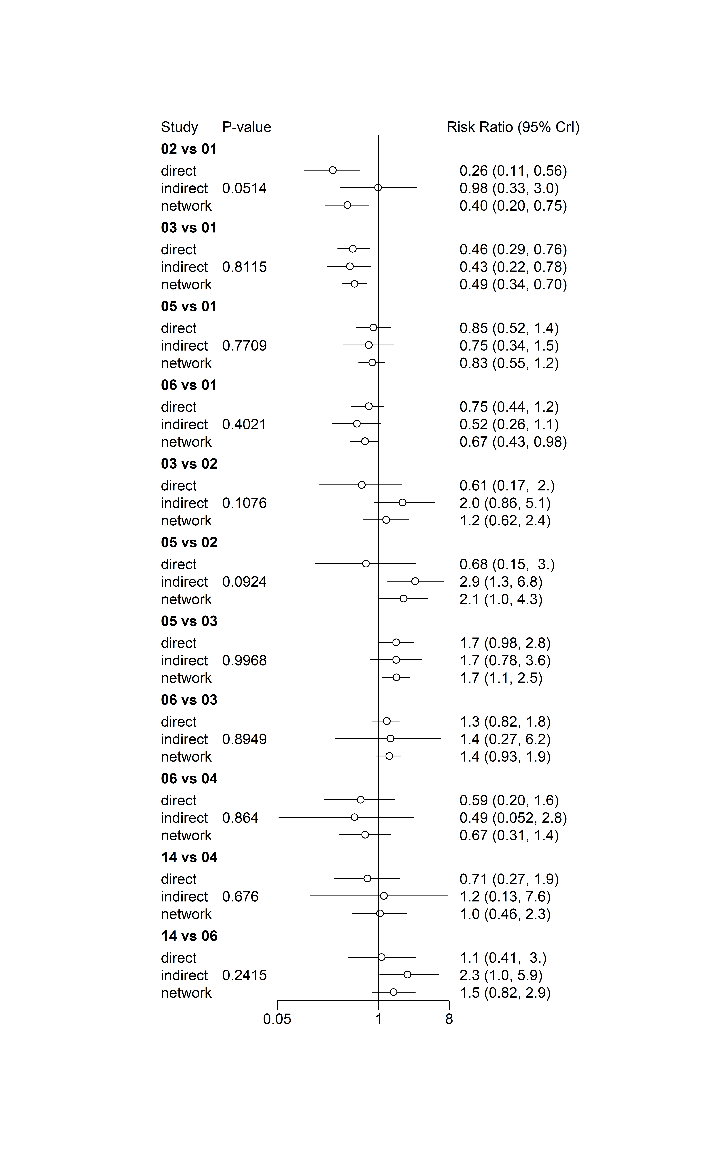


**9D**


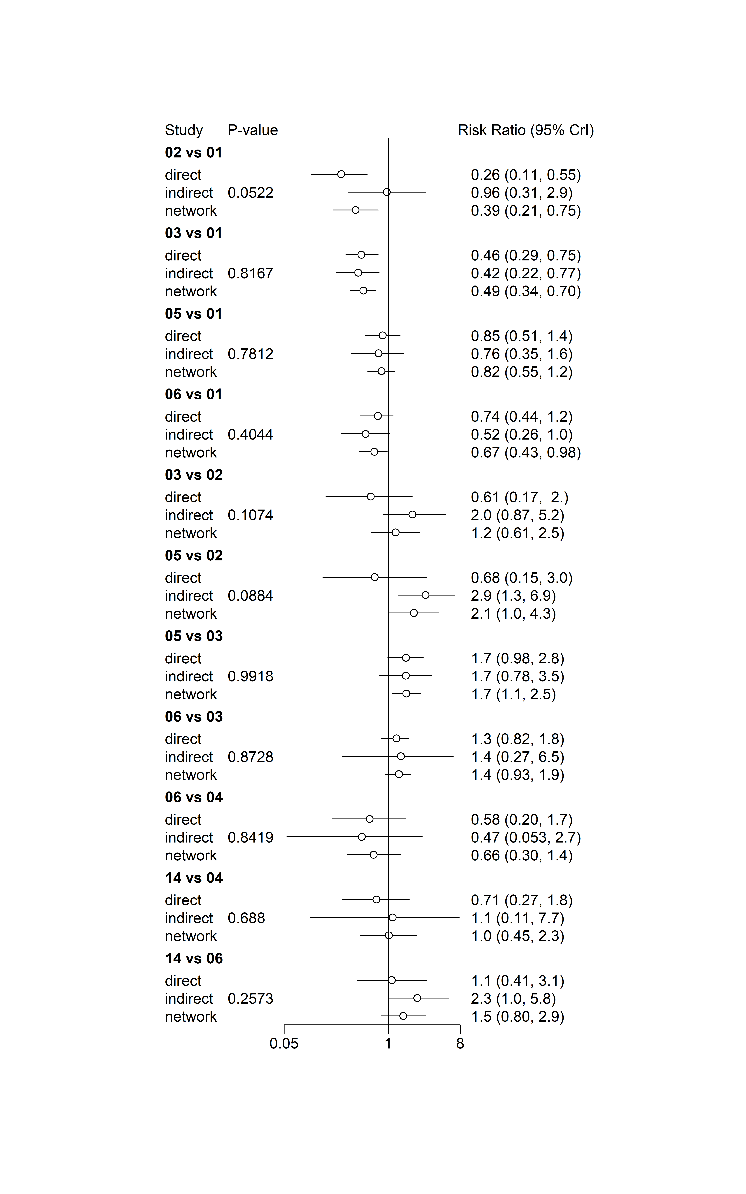


**9E**


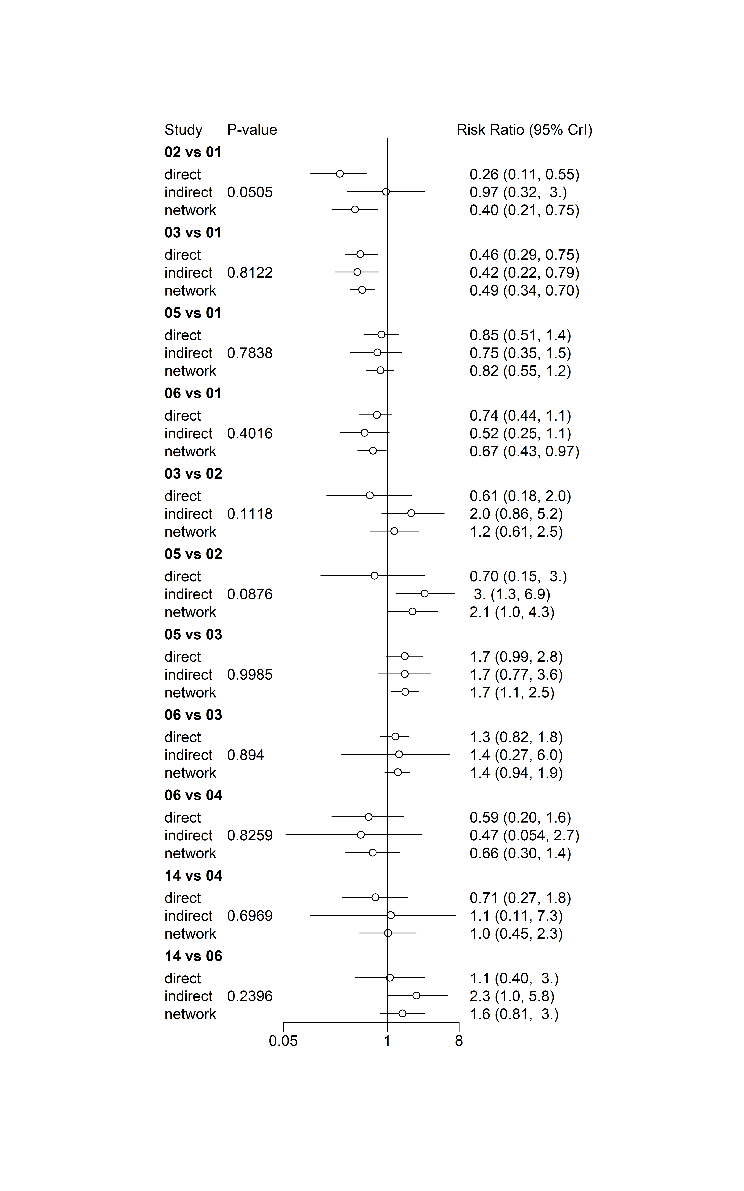

Supplement: Supplementary file 1 [file DataSheet1.docx]
